# Synthesis and Immunogenicity of Pseudo-Oligosaccharides Structurally Related to Repeating Units of Capsular Phosphoglycans of Human Pathogens †

**DOI:** 10.3390/molecules30153068

**Published:** 2025-07-22

**Authors:** Elena A. Khatuntseva, Anastasia A. Kamneva, Dmitry V. Yashunsky, Nikolay E. Nifantiev

**Affiliations:** N.D. Zelinsky Institute of Organic Chemistry, Russian Academy of Sciences, Leninsky Prospect 47, Moscow 119991, Russia; glyco@ioc.ac.ru (E.A.K.); nastyakamneva@icloud.com (A.A.K.); yashunsky1959@yandex.ru (D.V.Y.)

**Keywords:** phosphoglycans, phosphodiester linkage, capsular polysaccharides, synthesis, neoglycoconjugates, immunogenicity, conjugate glycovaccines, human pathogens

## Abstract

This review focuses on the synthesis of spacer-armed phosphooligosaccharides structurally related to the capsular phosphoglycans of pathogenic bacteria, including the *Haemophilus influenzae* serotypes a, b, c, and f, *Neisseria meningitidis* serogroups a and x, the *Streptococcus pneumoniae* serotypes 6a, 6b, 6c, 6f, 19a, and 19f, and the *Campylobacter jejuni* serotype HS:53, strain RM1221, in which the phosphodiester linkage is a structural component of a phosphoglycan backbone. Also, in this review, we summarize the current knowledge on the preparation and immunogenicity of neoglycoconjugates based on synthetic phosphooligosaccharides. The discussed data helps evaluate the prospects for the development of conjugate vaccines on the basis of synthetic phosphooligosaccharide antigens.

## 1. Introduction

Among many smart tools for the colonization of human organs, pathogenic bacteria use a multilayer cell envelope [1,2], which is largely composed of glycans and their conjugates. The outermost thick layer found in the majority of Gram-negative and some Gram-positive bacteria is called a glycan capsule, the composition and structure of which depend on the particular bacterial species [3]. Most capsular glycans are long-chain linear negatively charged CPSs or phosphoglycans. In the course of infection, this part of the bacterial outer shell first comes into contact with the components of innate and adaptive immunity. The anionic glycans of pathogenic bacteria shield them from the action of the components of the complement system, phagocytes and cationic antimicrobial peptides, which are secreted by immune and epithelial cells and destabilize the membrane of pathogenic bacteria [4,5]. Thus, it was established [6] that the phosphoglycan capsule of *Hib* prevents phagocyte attachment and the subsequent uptake of these bacteria. Moreover, *Hib* can survive and multiply after having been engulfed by a phagocyte in the acidic medium of phagolysosomes [7]. On the one hand, these biopolymers act as both protective shields and adhesive agents, enabling the bacterial evasion of host immunity [8], and on the other hand, they represent a target for the host immune system.

It is known that a key step in the adaptive immune response is the production of antigen-specific antibodies, and the presence of IgG indicates the development of immunological memory to surface biopolymers [9]. Immunological memory enables the body to quickly recognize the antigen on the surface of the pathogen during the second and subsequent contact and more effectively activate the body’s defenses. This phenomenon encouraged the development of a whole range of antibacterial prophylactic vaccines. To date, the most effective type of vaccines for the prevention of bacterial infections are conjugate vaccines [10,11,12,13,14,15,16], in which a capsular glycan of the targeted pathogen is covalently linked to a protein carrier. The use of glycoconjugates helps circumvent the problem of the low immunogenicity of CPS and directs immunity along the T-dependent pathway [17,18,19,20].

The majority of commercial conjugate vaccines are currently manufactured on the basis of CPS produced in bacterial cell cultures. This approach has significant disadvantages, which are the laborious and operationally challenging steps of manufacturing, sophisticated quality control, and the high cost of producing pathogenic microbiological material. At present, an advanced approach to glycan conjugate vaccines is being developed, which employs synthetic OS antigens as an alternative to bacterial CPS [21,22,23,24,25,26].

In the first step of conjugate OS vaccine development, it is important to specify the particular bacterial glycoantigen to be mimicked. The probability factor speaks in favor of regular glycans, which have a repeating unit. These include the CPS of both Gram-positive and Gram-negative bacteria, lipopolysaccharide *O*-antigens of Gram-negative bacteria, and the cell-wall teichoic acids and lipoteichoic acids of Gram-positive bacteria. Today, commercial glycoconjugate vaccines incorporate bacterial glycan antigens exclusively.

In the second step, the optimum length of the OS, which is sufficient for the induction of protective immunity, has to be specified. On the one hand, the use of shorter OSs can significantly reduce production costs, and on the other hand, the OS chain has to be long enough to mimic the polymer and minimize the possible influence of the terminal monosaccharide residue. Identification of the minimum length of the OS antigen is performed in laboratory animals, which are immunized with the conjugates of synthetic oligosaccharides of different lengths, and the interaction of induced antibodies with bacterial antigens or directly with bacteria is investigated. It is commonly accepted [27] that the minimum length of an effective glycoantigen is 3–4 repeating units. A reliable algorithm that can predict the optimum length of OS antigens has not yet been developed. Today, the identification of protective glycotopes for conjugate vaccine candidates can be defined by the screening of glycan arrays, which encompass a series of synthetic OSs related to capsular glycans [28,29] in combination with conformational studies [29,30]. Alternatively, the development of the anti-Hib vaccine Quimi-Hib^®^ (Centro de Ingeniería Genética y Biotecnología (CIGB), Republic of Cuba), which is a unique commercial synthetic OS-based conjugate vaccine, circumvented the problem of the choice of the optimum antigen length by the production of a protein-conjugated homologous phosphooligosaccharide obtained by oligomerization, with 7–8 repeating units as the average length of antigens [31]. The choice of the optimum structure of an OS antigen for a glycovaccine is complicated by the possibility of structural changes in a synthetic antigen after the injection of the vaccine into the recipient’s body. Partial lysis or migration of acetyl groups can occur in lymphs (pH 7.4–9.0) or endolysosomes of follicular B-lymphocytes [32,33,34], which are known to have an acidic environment.

**Figure 1:**
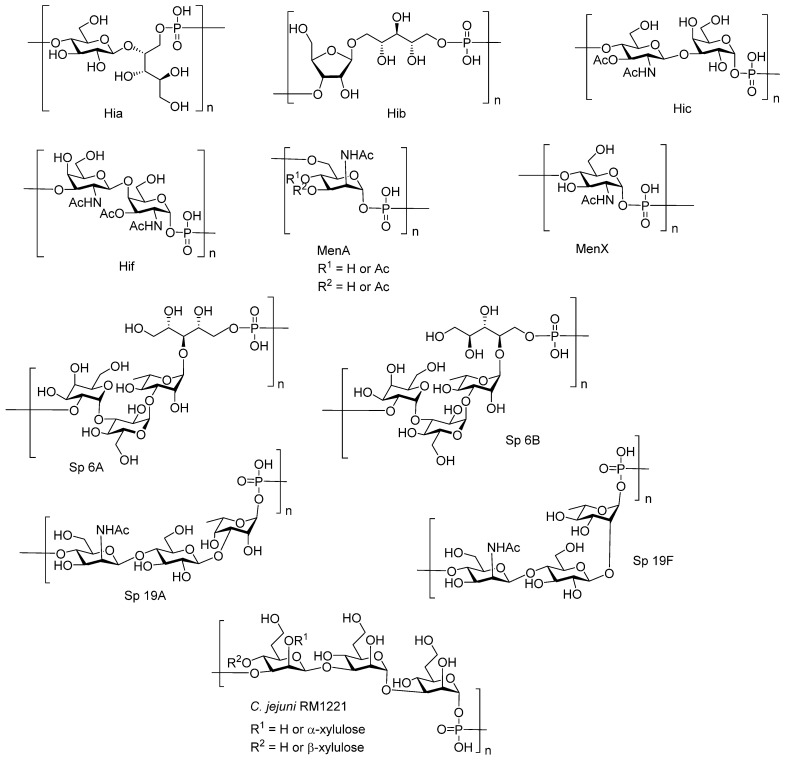
Examples of bacterial capsular phosphoglycans.

In a number of human pathogens, a phosphodiester bond is involved in the formation of the main polymer chain. Phosphoglycans of this type were found, for example, in the cell wall of the human pathogens *Hia*, *Hib*, *Hic*, and *Hif*, the *S. pneumonia* serotypes 6A, 6B, 11A, 17F, 19A, and 19F, the *N. meningitidis* serogroups *A* and *X*, the *Campylobacter jejuni* serotypes HS53 (strain RM1221) and HS1 (strain ATCC 43429), and *Escherichia coli* K100 and K2 (Figure 1) [35,36,37,38]. Today, eleven bacterial pathogens of this type (*Hib*, *Men A*, and the *S. pneumonia* serotypes 6A, 6B, 10A, 11A, 15B, 18C, 19A, 19F, and 23F) are targeted by preventive vaccination, with commercial conjugate vaccines based on the corresponding capsular phosphooligosaccharides [21].

In this review, we focus on the synthesis of spacer-armed mono-, di-, and oligomeric antigens structurally related to bacterial capsular phosphoglycans (Figure 1), in which the repeating units are connected via a phosphodiester bond, and consider the preparation and immunogenicity of neoglycoconjugates on the basis of these antigens.

**Figure 2:**
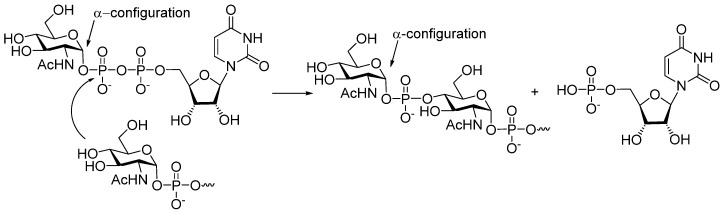
Biosynthesis of *MenX* phosphoglycan involves nucleotide glycosyl donor uridine-5′-diphosphate-*N*-acetyl glucosamine and is catalyzed by hexose phosphotransferase [41].

Phosphoglycans are the common glycocalyx components of pathogenic Gram-negative and Gram-positive bacteria [35,36,39], yeast [39], and protozoan parasites [39,40]. In a living cell, phosphodiester-linked carbohydrates are arranged via the transfer of a hexose 1-phosphate to a glycan acceptor. In the presence of enzymes, which belong to the Stealth enzyme family, a hydroxyl group of a glycan acceptor attacks the phosphoester group in the nucleotide donor, and finally, a phosphodiester interglycosidic bridge unit is formed. It was established [41] (Figure 2) that in *MenX* bacteria, the hydroxyl group of *N*-acetyl glucosamine acceptor attacks the P-atom of uridine-5′-diphosphate-*N*-acetyl glucosamine to form an α-glycosyl phosphodiester, and the uridine monophosphate moiety serves as a leaving group.

A similar method of the establishment of a phosphodiester intersaccharide bridge is used in laboratory practice for the preparation of phosphooligosaccharides related to bacterial phosphoglycans. For the efficient and stereoselective formation of a phosphodiester linkage, a phosphodiester synthon is first introduced into one of the saccharide blocks, and the resulting product is reacted with a free hydroxyl group of another saccharide block.

**Figure 3:**
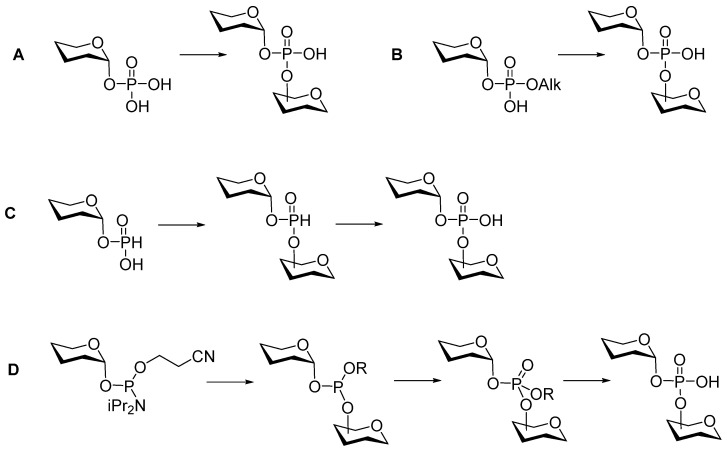
Three main types of synthetic approaches used in the preparation of glycosyl phosphodiesters.

As a rule, phosphodiesters, along with their diverse precursors (mono- and diphosphates, H-phosphonates, and phosphamidites; Figure 3), decompose under the conditions of a glycosylation reaction. Therefore, the retrosynthetic analysis of phosphoglycans suggests the formation of phosphodiester bridges within the latest steps of the synthetic route after the glycosidic linkages are already established. Usually, the preparation of phosphooligosaccharides related to natural biopolymers is a multi-step synthesis, which can be performed following a linear, convergent, or oligomerization pathway via the formation of an O-P-O tether between selectively protected and activated saccharide blocks.

The conventional synthetic blocks for the preparation of glycosylphosphodiesters are monophosphates (Figure 3A), diphosphates (Figure 3B), H-phosphonates (Figure 3C), and phosphoramidites (Figure 3D). Previously developed methods of condensation involved phosphorus (V) chemistry (Figure 3A,B) and were promoted by *N*,*N*′-dicyclohexylcarbodiimide and 1-(2,4,6-triisopropylbenzenesulfonyl)-3-nitro-1H-1,2,4-triazole. In the late 1980s, methods A and B gave way to fast, efficient, and convenient techniques that employed H-phosphonates (Figure 3C) and phosphoramidites (Figure 3D).

The H-phosphonate condensation general procedure [42,43] was first proposed in the 1950s for oligonucleotide synthesis by Todd et al. [44], and it was further developed [45,46] and adapted for solid-phase synthesis [47] and customized to the needs of phosphoglycan chemistry [39,48]. The phosphoramidite method was first proposed by van Boom [49] and was later successfully applied in the solid-phase preparation of long-chain oligophosphodiesters [50] and the P-modified analogs of glycosylphosphate oligomers [51]. In addition to current methods, novel approaches are being actively developed, which are aimed at the preparation of glycosylphosphates with a predetermined anomeric configuration and the synthesis of the stabilized mimetics of phosphoglycans [52,53,54,55,56,57,58].

One of the key features of bacterial phosphoglycans and synthetic phosphooligosaccharides is their susceptibility to degradation via hydrolytic cleavage, transesterification, and rearrangement. Thus, PRP (Figure 1) was found to degrade spontaneously [59,60] in aqueous media. This molecule is destabilized by a hydroxyl group at C-2 (D-ribose), which is located in close proximity to the phosphodiester moiety and promotes the depolymerization and formation of cyclophosphate and phosphate monoester terminal groups [61]. In vaccine production, the inherent tendency of PRP to autolyze results in the loss of manufactured phosphoglycan and conjugate preparations [60]. Additionally, the low stability of PRP imposes significant limitations on the use of liquid Hib vaccines, especially in view of the acceleration of the degradation process in the presence of an alum adjuvant [62]. Stability studies conducted by Cintra et al. [60] showed that the rate of PRP depolymerization accelerates substantially with an increase in pH in the range 5.41–7.55 and with a rise in temperature in the interval of 28–40 °C. As a result, it may be assumed that in a host organism, PRP intensely degrades into fragments, which neutralize anti-PRP protective antibodies, thus hampering the immune response. In a similar way, partial PRP destruction after immunization with a conjugate *Hib* vaccine may result in the loss of Hib epitopes and reduce the level of protective anti-Hib antibodies. Two more factors that affect the stability of PRP are the presence of Na^+^ [60] and Ca^2+^ [59] cations. Similar to PRP, the capsular phosphoglycan of *Hif* (Figure 1) was shown to decompose under mild conditions [59].

MenA capsular phosphoglycan is especially susceptible to hydrolysis. It is assumed that the hydrolytic destruction of α-glycosylphosphodiester linkage occurs by two pathways. One of these includes the formation of an oxocarbenium ion, and within another pathway, the phosphodiester bond is cleaved with the assistance of the axial NAc group located at C-2 of the ManNAc, and a thermodynamically stable oxazoline is formed [63].

## 2. Synthesis of Pseudo-Oligosaccharides Structurally Related to PRP

**Figure 4:**
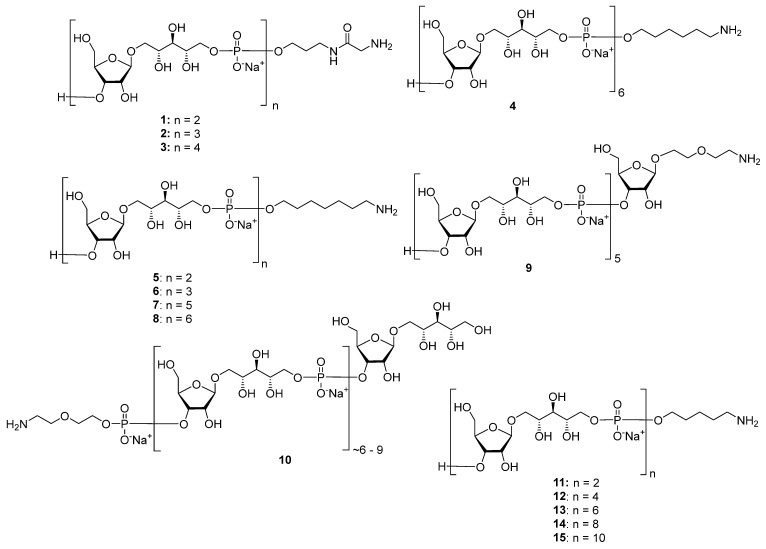
Synthetic pseudo-oligosaccharides structurally related to PRP.

Highly effective infant immunization with conjugate *Hib* vaccines, which are composed of a partially depolymerized *Hib* capsular phosphoglycan and a protein carrier (see review [15]), inspired researchers to develop conjugate vaccines using oligomer synthetic PRP fragments (Figure 4). Several series of PRP-related oligomers were synthesized, which were equipped with different types and locations of amino-spacers for convenient attachment to a protein carrier (compounds **1**–**15**, Figure 4).

In pseudo-oligosaccharide **13** with a *N*-glycyl-aminopropyl spacer, which comprises two, three, or four residues of PRP repeating units [64], as well as in hexamer **4** [65] and oligomers **5**–**8** [66], the ω-aminospacer is linked to the D-ribose fragment via a phosphodiester bond. In pentamer **9** [67], the spacer is attached immediately to C-1 of a D-ribose residue as an aglycone. In the oligomeric mixture **10** [68] and in most of the representative oligomer series, which comprises the individual compounds **11**–**15** [69], the spacer is connected to a D-ribose via a phosphodiester bridge. In this review, basic synthetic blocks and strategies used in the preparation of compounds **1**–**15** are briefly considered, with a focus on the arrangement of a phosphodiester linkage and the introduction of a ω-aminospacer. A more detailed consideration of the syntheses of pseudo-oligosaccharides **1**–**15** was presented in our earlier review [15].

**Scheme 1:**
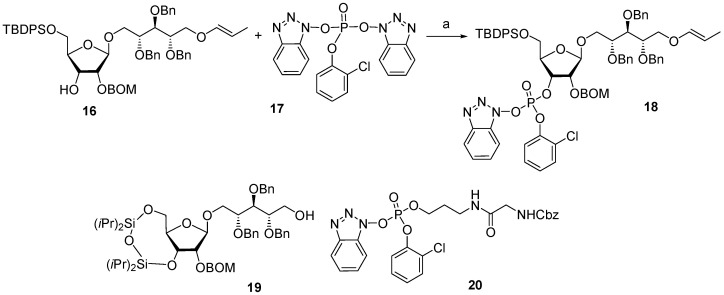
Key building blocks for the synthesis of spacer-armed PRP-related oligomers (**1**–**3**). Reagents and conditions: (**a**) Py/dioxane, r.t., 30 min [64,70,71].

Between 1988 and 1992, van Boom and his research group at Leiden University synthesized spacer-armed PRP-related fragments for the first time. Dimer **1** [64,70], trimer **2** [64,70], and tetramer **3** [71] (Figure 4) were prepared by a sequential elongation of the oligomer chain from the non-reducing end, and the final equipment with an aminospacer. In brief, the phosphorylation of a free 3-OH group in the selectively protected riboside **16** with bis(benzotriazol-1-yl) (2-chlorophenyl) phosphate **17** produced the key activated phosphotriester building block **18** (Scheme 1). The condensation of compound **18** with the selectively protected disaccharide **19** in the presence of *N*-methylimidazole in Py, followed by the removal of the 1-*O*-propenyl group, was carried out in a sequential manner, one, two, or three times, followed by the final capping with phosphotriester **20**. Total deprotection afforded the series of conjugation-ready compounds **1**–**3**.

**Figure 5:**
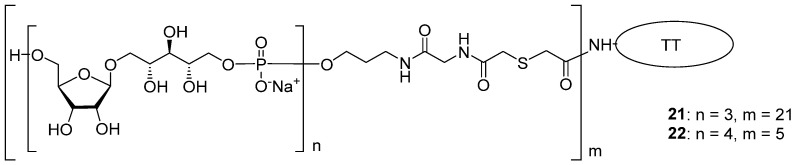
Conjugates **21** and **22** obtained from PRP oligomers **2** and **3** with TT [71].

The spacer-armed trimer **2** and tetramer **3** were conjugated to TT (Figure 5) to create the corresponding neoglycoconjugates **21** and **22**. The antigenic properties of oligomers **2** and **3** as components of neoglycoconjugates **21** and **22** were examined [71] in competitive inhibition ELISA experiments. A conjugate of bacterial PRP and tyramine was used as a coating antigen, and normal human serum and bacterial PRP were used as positive controls.

Total inhibition was observed when bacterial PRP and conjugate **22** (tetramer + TT, Figure 5) at a concentration of 25 μg/mL were used as inhibitors, and conjugate **21** with the trimeric PRP–antigen was less effective. Compared to conjugates, the inhibitory capacity of the unconjugated oligomers **2** and **3** was much lower, and at a concentration of 25 μg/mL, it did not reach 40%.

The immunogenicity of glycoconjugates **21** and **22** was studied [71] in mice at a dose of 1 µg of phosphoglycan per mouse. IgG antibodies in immune sera were analyzed in ELISA using a bacterial PRP–tyramine conjugate as a coating antigen and normal human serum as a positive control. Conjugate **21** with a trimeric antigen was found to be low immunogenic in a mouse model. In IgG ELISA, the mean optical density value for the sera of immunized mice was less than two times that of the corresponding value obtained for the sera of mice that received PBS.

For the immune sera from mice vaccinated with conjugate **22**, which comprised tetrameric residues, the mean optical density value was three times that of the negative control, thus unambiguously evidencing the immunogenicity of conjugate **22**. Immunization experiments were conducted using preparations formulated with AlPO_4_ or without an adjuvant. It was shown that the presence of AlPO_4_ had no effect on the result of immunization [71].

To date, there is no animal model that reliably predicts the immunogenicity of glycoconjugate preparations in humans, and the choice of experimental animals for the examination of the immune activity of *Hib* preparations is usually at the discretion of the researcher. In the blood sera of immunized mice and guinea pigs, the concentration of antibodies directed against *Hib* antigens does not reach 1 mg/mL, and these animals are not recommended for laboratory studies of conjugate vaccines [66].

**Scheme 2:**
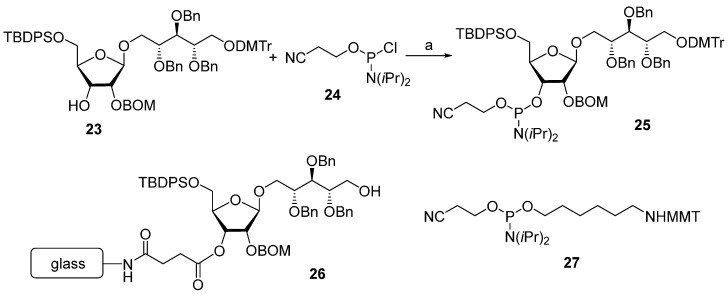
Key building blocks for the synthesis of hexamer **4**. Reagents and conditions: (**a**) diisopropylethylamide/dichloromethane, 85% yield [65].

As shown in Scheme 1, the key reaction in the assembly of the synthetic *Hib* phosphooligosaccharides **1**–**3** is the arrangement of the O-P-O linkage, which can be applied iteratively. As no new chiral centers or other types of isomerism are created, this reaction sequence can be fulfilled using a solid-phase approach. This approach significantly decreases the number of laborious steps of the chromatographic separation of the product. In 1989, van Boom synthesized the spacer-armed hexamer **4** using controlled-pore glass as a solid support [65]. First, the selectively protected pseudo-disaccharide **23** was phosphitylated with chlorophosphoramidite **24** (Scheme 2), and the resulting phosphoramidite **25** was used for a stepwise extension of the oligomer chain starting from ribosylribitol **26**, which was immobilized on a glass support. After each step of chain elongation, the phosphorus atom was oxidized with iodine in an acetonitrile/water/collidine mixture to obtain the corresponding phosphotriester, and the terminal DMTr protective group was removed to provide a free hydroxyl group for the next phosphitylation step. After the sequential attachment of five repeating units and spacer **27** and the removal of the protective groups, the target spacer-armed hexamer **4** was obtained.

**Scheme 3:**
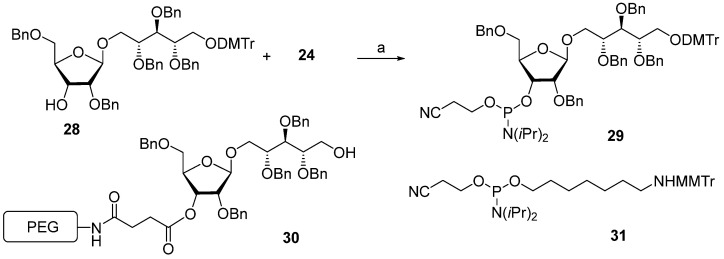
Key building blocks used in the synthesis of the spacer-armed oligomers **5**–**8**. Reagents and conditions: (**a**) 1H-tetrazole/acetonitrile [66].

In 1992, Kandil et al. [66] synthesized the series of spacer-armed Hib oligomers **5**–**8** on a PEG support using a strategy similar to the synthesis of hexamer **4** (Scheme 3). In this synthesis, the tert-butyldiphenylsilyl and benzyloxymethyl protective groups in the starting pseudo-disaccharide **28** were replaced with easily removable benzyl groups. Thus, the sequential condensation of phosphoramidite **29** with PEG-immobilized pseudo-disaccharide **30** and detritylation was repeated five times. Next, the pre-spacer **31** was attached, and finally, total deprotection resulted in the formation of hexamer **6**. The latter was conjugated to synthetic peptides structurally related to the *Hib* outer membrane protein, and one of these preparations showed immunogenicity comparable to that shown by the commercial *Hib* vaccine [72].

**Scheme 4:**
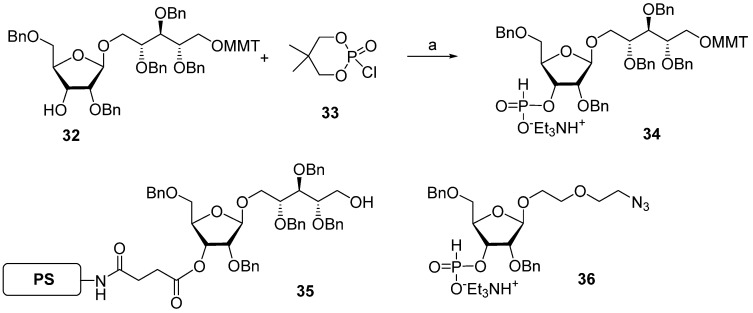
Key building blocks for the synthesis of pentamer **9** [67]. Reagents and conditions: (**a**) H_3_PO_3_, Py, yield of 84%.

The same year, a group of Swedish researchers under the leadership of Norberg published [67] the synthesis of the *Hib* pentamer **9** on a polystyrene support (Scheme 4). In this work, the H-phosphonate method was used for the establishment of a phosphodiester bond, which subsequently became the most popular in the preparation of oligophosphoglycans related to the glycocalyx components of bacteria and parasites [39,56]. The selectively protected disaccharide **32** was reacted with 5,5-dimethyl-2-chloro-1,3,2-dioxaphosphorinane 2-oxide (compound **33**) in Py and the presence of phosphonic acid to obtain H-phosphonate **34** with an 84% yield.

The activation of H-phosphonates for subsequent attachment to alcohols can be achieved in the presence of the acyl chlorides of sterically hindered acids, e.g., PivCl or 1-adamantanecarbonyl chloride. In this reaction, the formation of by-products depends significantly on the order of the addition of reagents. For example, the activation of H-phosphonate by PivCl in Py in the absence of alcohol led to the formation of unwanted bis-acylated phosphites, and the use of a large excess of PivCl in solid-phase synthesis resulted in the pivaloylation of the support [67]. The search for the optimum conditions for the attachment of H-phosphonate **34** to be selectively protected and immobilized on the solid support monomer **35** resulted in the discovery of the most efficient proportion—5 eq. of PivCl and 5 eq. of H-phosphonate **34**—that provided the adduct with a 95% yield. The authors noted [67] that the yield decreased with each chain extension step and dropped to 86% in the fourth cycle. The optimum sequence of reagent addition was found as follows: in the first step, H-phosphonate was activated by the addition of PivCl in Py, and then, the mixture was added to the immobilized alcohol. As a result, the yield of the pentamer increased to 96%. In the final step, the chain was capped with 2-(2-azidoethoxy) ethyl riboside **36**, H-phosphonate was oxidized with iodine in 2% aqueous Py, and the protective groups were removed to obtain oligomer **9**.

**Scheme 5:**
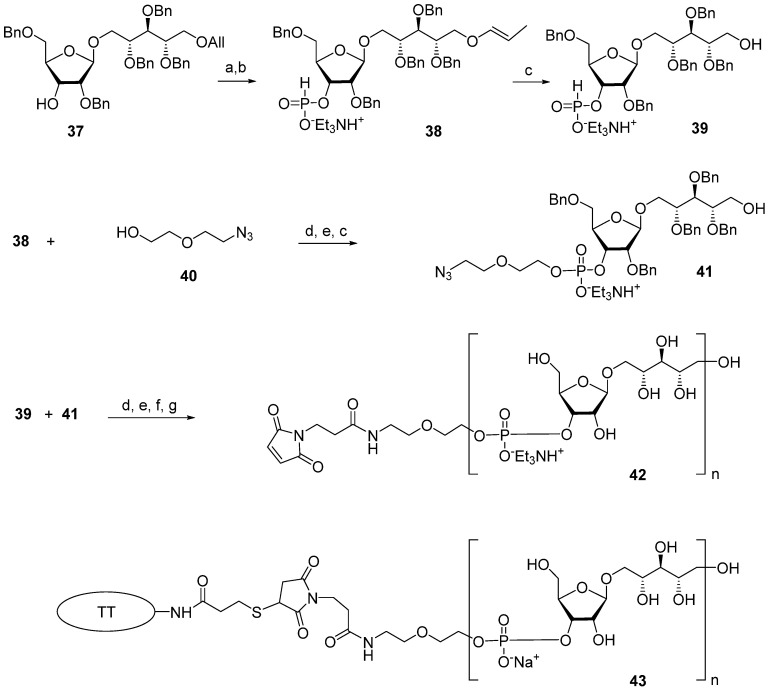
Key building blocks for preparation of oligomeric mixture **10** (n= 6–9) [68]. Reagents and conditions: (**a**) potassium tert-butoxide/DMSO, 100 °C; (**b**) PCl_3_, imidazole, Et_3_N; (**c**) CH_3_COOH/H_2_O, 80 °C; (**d**) PivCl/Py; (**e**) I_2_, Py/H_2_O; (**f**) H_2_, Pd/C, EtOH-H_2_O-EtOAc-AcOH at 1.5 atm; (**g**) SMP.

The multistep synthetic pathways discussed above are based on the linear sequential elongation of an oligomeric chain. As a rule, laboratory processes of this type cannot be scaled up to a commercial scale because of the high cost. For profitable manufacturing, a convergent synthetic scheme is needed. Under the leadership of Verez-Bencomo and Roy, a convergent synthesis of a mixture of homologous PRP oligomers (Figure 4, compound **10**) was developed and then scaled up to the commercial production of the anti-*Hib* vaccine Quimi-Hib^®^ (Heber Biotec, S.A., Republic of Cuba). In this synthesis, polycondensation of a bifunctional monomer is employed for the establishment of O-P-O linkages as a key step of oligomer synthesis in the place of stepwise elongation (Scheme 5).

In brief, the allyl group of the selectively protected pseudo-disaccharide **37** is isomerized into the 1-*O*-propenyl group by the action of potassium tert-butoxide in DMSO. The resulting isomer is converted to H-phosphonate **38** by the action of PCl_3_ and imidazole, and finally, the 1-*O*-propenyl group is removed under acidic conditions to obtain the key heterobifunctional monomer **39**. The interaction of **38** with the diethylene glycol spacer **40** in the presence of PivCl/Py, followed by oxidation with iodine in aqueous Py, and the further removal of the 1-*O*-propenyl group, creates alcohol **41**, which serves as a terminal unit in the polycondensation. The polycondensation of **39** and **41** is carried out in the presence of PivCl in Py, followed by oxidation of the mixture of H-phosphonates into the corresponding phosphodiester mixture, reduction of the azido groups, *N*-acetylation, and total deprotection. The obtained mixture of the spacer-equipped oligomer **10** is activated by the action of SMP to obtain a conjugation-ready mixture of maleimide **42**. The conjugation of the activated oligophosphodiesters with thiolated TT results in the production of a set of conjugate **43** with the average weight ratio PRP:TT of 1:2.6 [31,68].

On the basis of the mixture of conjugate **43**, an anti-*Hib* vaccine, Quimi-Hib^®^, was developed, which successfully passed all the required preclinical [73] and clinical trials [31,74]. The antigenicity of synthetic PRP oligomer **10** was compared to the antigenicity of bacterial PRP in ELISA experiments [31]. A conjugate of a mixture of the spacer-equipped oligomer **10** with HSA (**10**-HSA), prepared similarly to conjugate **43**, and a conjugate of partially depolymerized bacterial PRP (PRPDp30) with HSA were used as coating antigens. The PRPDp30-HSA conjugate was obtained by the reductive amination of the mixture of bacterial PRP depolymerized via periodate oxidation to a length of ~30 monomeric units and conjugation with HSA [68]. Standard rabbit anti-PRP antibodies were obtained by the immunization of animals with two licensed commercial conjugate vaccines based on bacterial PRP. These were Hiberix^®^ (PRP-TT), which contains PRP activated by cyanogen bromide and conjugated to TT via an adipic acid dihydrazide linker, and Vaxem-Hib^®^ (PRP-CRM197), which is a product of the conjugation of partially depolymerized bacterial PRP with an avDP10 repeating unit and the protein carrier CRM197 via an adipic acid dihydrazide spacer. The correlation coefficient for the titers obtained in the ELISA experiments with standard rabbit sera and the coating antigens 10-HSA and PRPDp30-HSA was in the high-value range (0.972–0.978), indicating the similarity of the antigenic properties of the synthetic oligosaccharide **10** and *Hib* CPS [75].

For the preliminary evaluation of the immunological activity of the conjugate **43** in vivo, rats, mice, and rabbits were chosen as experimental animals. Rats were immunized subcutaneously twice at an interval of 4 weeks with a dose of conjugate **43** containing 2 µg of the ligand **10** [75]. Mice were immunized intraperitoneally three times at an interval of 2 weeks with a dose of conjugate **43** containing 2.5 µg of ligand **10**. Rabbits were immunized with conjugate **43** using both two-step and three-step regimens at a dose of 5 µg of ligand **10** per animal. The efficiency of immunization was assessed by the evaluation of IgG titers, which were calculated as the logarithm of the highest dilution at which the light absorbance of the diluted serum sample is twice as high as that of the negative control. For the negative control, the animals were injected with PBS. A PRP-HSA conjugate was used as a coating antigen in the ELISA experiments. The study of the PRP-specific antibodies showed that the immune response to conjugate **43** in rodents was weak and inconsistent. In contrast, the immunization of rabbits with conjugate **43** efficiently evoked PRP-specific antibodies, as shown in the inhibition ELISA experiments with bacterial PRP as the inhibitor [75].

In a phase 1 clinical trial [31], more than 100 children aged 4 to 5 years were immunized with a single dose of a vaccine preparation formulated on the basis of conjugate **43** without an adjuvant. Comparative studies of the immunogenicity of synthetic antigens in conjugate **43** and partly depolymerized bacterial PRP in Vaxem-Hib^®^ showed that the average anti-PRP IgG titers were similar. It was found that PRP-specific IgG antibodies induced in children by immunization with conjugate **43** had bactericidal activity comparable to that stimulated by the action of Vaxem-Hib^®^. In the course of phase 2 clinical trials [31], 1141 infants were immunized three times at 2, 4, and 6 months of age with or without AlPO_4_, and the positive control group was immunized with Vaxem-Hib^®^. Antibody tests showed that 99.7% of infants had serum anti-PRP IgG concentrations >1 µg/mL, which is known to provide prolonged protection against Hib infection [76], and the mean concentration of anti-PRP IgG was as high as 27.4 µg/mL.

It is noteworthy that in Quimi-Hib^®^, different-sized oligomeric antigens were present, thus circumventing the problem of the elucidation of the optimal length of an oligomeric PRP antigen. The success of the development and application of the cost-effective vaccine Quimi-Hib^®^ with a synthetic *Hib*-antigen inspired scientists to search for the shortest protective *Hib*-antigen, which is likely to be in the range of chain lengths that are obtained during the polycondensation of **39**.

**Scheme 6:**
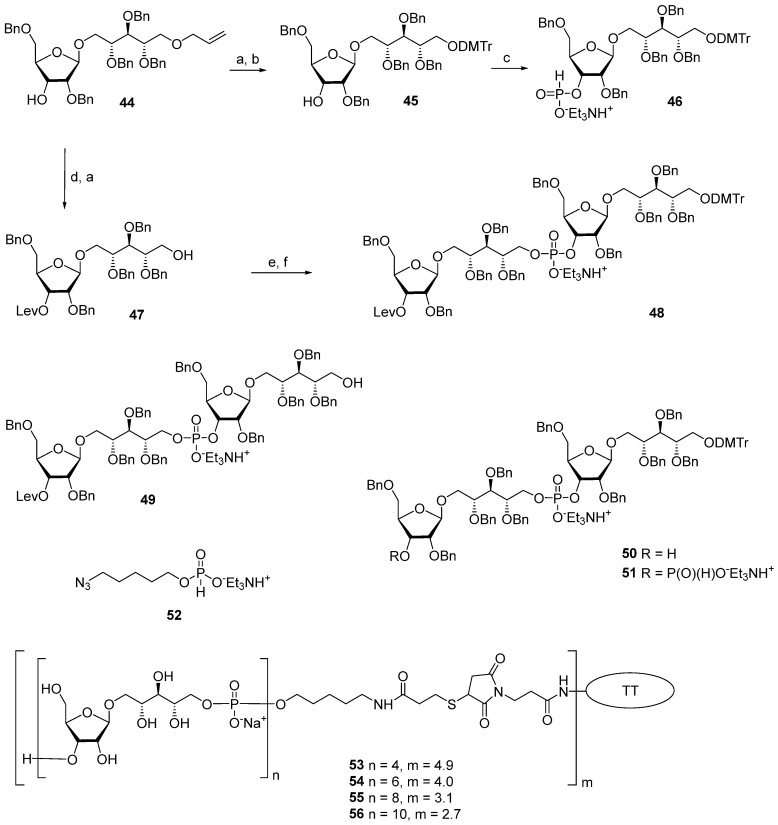
Key building blocks for preparation of oligomers **12**–**15 [69]**. Reagents and conditions: (**a**) Pd(PPh_3_)_4_, 1,3-dimethylbarbituric acid, methanol, 75%; (**b**) DMTCl, DMAP, CH_2_Cl_2_, 93%; (**c**) PCl_3_, Et_3_N, imidazole, CH_2_Cl_2_, 0 °C, 85%; (**d**) levulinic acid, DMAP, CH_2_Cl_2_, 93%; (**e**) **46**, PivCl, Py, 0 °C; (**f**) I_2_, Py/H_2_O, 85%.

One of the novel and efficient approaches to the preparation of PRP-related oligomeric phosphoglycans suggested by Seeberger et al. [69] involved the elongation of the chain by two PRP repeating units at once (Scheme 6). The applied strategy effectively shortened the task of the oligomer assembly and raised the degree of convergence.

For the preparation of a selectively protected and activated bifunctional dimeric key unit, the universal precursor **44** was deallylated, and the resulting diol was regioselectively 4,4′-dimethoxytritylated at the primary hydroxyl group to obtain compound **45**, which was then converted to H-phosphonate **46** by the action of PCl_3_, Et_3_N, and imidazole. For the preparation of alcohol **47**, the universal precursor **44** was levulinoylated and deallylated. Alcohol **47** was reacted with H-phosphonate **46** in PivCl/Py to obtain the key dimeric precursor **48** with readily removable orthogonal levulinoyl and dimethoxytrityl protective groups. The de-4,4′-dimethoxytritylation of pseudo-tetrasaccharide **48** in the presence of trichloroacetic acid afforded alcohol **49** with a free terminal hydroxyl group in the ribitol residue. The delevulinoylation of universal precursor **48** by the action of hydrazinium acetate resulted in the formation of alcohol **50**, which was converted into H-phosphonate **51**. The combination of H-phosphonate **51** and primary alcohol **49**, followed by oxidation, yielded a tetramer, which was, in turn, de-4,4′-dimethoxytritylated and reacted with H-phosphonate **46** for the elongation of the chain or with the 5-azidopentyl derivative **52** for chain termination. Using this elegant strategy, PRP-related tetramer (85%), hexamer (85%), octamer (83%), and decamer (80%) were prepared, which were subjected to sequential detritylation, the attachment of a 5-azidopentyl spacer under the action of H-phosphonate **52**, the transformation of the azido group into an amino group, and total deprotection to obtain the spacer-armed oligomers **12**–**15** (Figure 4). After activation, oligomers **12**–**15** were reacted with TT to obtain conjugates **53**–**56** [69].

The antigenicity of oligomers **11**–**15** (Figure 4) was examined using a glycan microarray. Compounds **11**–**15** were immobilized on a *N*-hydroxysuccinimide hydrogel surface on glass slides and reacted with polyclonal anti-*Hib* hyperimmune rabbit sera or standard human sera, which were mixed with different concentrations of the WHO PRP standard as an inhibitor. The subsequent addition of a fluorescent secondary antibody revealed the dependence of the fluorescence intensity on the PRP concentration, thus proving the presence of antibodies specific to the synthetic PRP-oligomers **11**–**15**. Hexamer **13**, octamer **14**, and decamer **15** interacted with standard human sera in a similar way, binding with tetramer **12** was weaker, and in the case of dimer **11**, the adsorption of the antibodies was even less effective. In contrast, rabbit hyperimmune sera interacted with all five oligomers equally well [69].

The immunogenicity of conjugates **53**–**56** was studied using an animal model. Rabbits were immunized with these conjugates at a dose of 5 μg of an oligomer without an adjuvant, and the positive control group received ActHib^®^ (PRP-TT). Bacterial PRP was used as a coating antigen. Immunization with conjugates **53** and **55**, based on tetramer **12** and octamer **14** ligands, respectively, resulted in a high level of PRP-specific antibodies. Conjugates **54** and **56**, which comprised hexamer **13** and decamer **15**, induced substantially lower levels of PRP-specific antibodies [69]. Therefore, in a series of conjugates equipped with antigens with an even number of repeating units, the tetramer was the most promising synthetic antigen candidate for the design of an anti-*Hib* vaccine.

In a continuation of this work, Seeberger et al. synthesized [77] a series of mimetics of synthetic PRP-related dimer, tetramer, hexamer, and octamer compounds (**11**–**14**), which comprised 2-deoxy-, 2-deoxy-2-fluoro-, 2-deoxy-2-(*N*,*N*-dimethyl)-carbamoyloxy-, and 2-O-methylated ribose residues in different combinations. This research was aimed at the preparation of analogs of PRP antigens with higher hydrolytic stability. The study of the conjugates of these antigens with the protein carrier CRM197 showed that the most promising are mimetics, in which ribose residues are methylated at the position O-2. Conjugates of these mimetics with CRM197 stimulated Hib-specific immune responses in both animals and humans, which confirms the possibility of their commercial use in anti-*Hib* vaccines. The replacement of the 2-OH group, which promotes the hydrolysis of the phosphodiester bond, with a methoxy group allowed for a significant increase in the integrity of the antigen [77].

## 3. Synthesis of Oligomers Structurally Related to Capsular Phosphoglycans of Hia, Hic, and Hif

*Hib* bacteria are the common and most dangerous serotype of the *H. influenzae* species, which comprises at least five more encapsulated serotypes and numerous non-capsulated (non-typeable) variants. The introduction of anti-*Hib* conjugate vaccines [13,15,21,23] in national immunization schedules in a number of European countries, North and South America countries, Australia, and the South African Republic significantly contributed to a reduction in invasive diseases caused by *Hib*-infection [78,79]. At the same time, along with the expansion of anti-*Hib* vaccination programs, the spread of other serotypes of *H. influenzae* was reported [80,81,82,83,84,85,86,87,88,89]. As a consequence, after more than thirty years’ use of anti-Hib conjugate vaccines, an “antigenic shift” is observed, which is expressed in the increased incidence of invasive diseases, including meningitis, meningoencephalitis, and septicemia caused by encapsulated *H. influenzae* serotypes different from b [82,90].

In European countries, the number of invasive diseases caused by *Hia* is also growing. For example, in England, in 2022, Hia was responsible for 19% of all invasive disease cases caused by encapsulated *H. influenzae*. Before 2017, only sporadic cases occurred [80], and the contribution of *Hif* and *Hie* is also growing [90]. In South Africa, the rise of *Hie* is observed [82]. In American countries, *H. influenzae* diseases are often caused by *Hif* [82,91]. Also, an increase in invasive diseases caused by *Hia* [85,89,92,93] and *Hic* [91] was reported.

In the early 1990s, in the northern regions of Canada and Alaska, a routine anti-*Hib* immunization schedule was introduced, and in the late 1990s, a growing number of cases of *Hia* infection were registered. The majority of cases (more than 60%) were reported in children under five years of age. As a result, special research is being conducted in Canada aimed at the development of a vaccine for the prevention of diseases caused by *Hia* [84]. Considering that invasive diseases caused by encapsulated *H. influenzae* serotypes different from serotype b remain relatively rare and are often localized in specific areas, corresponding vaccines will have limited use according to epidemic indications. In this situation, conjugate vaccines with synthetic antigens structurally related to *H. influenzae* capsular glycans may be considered as a drug of choice.

**Figure 6:**
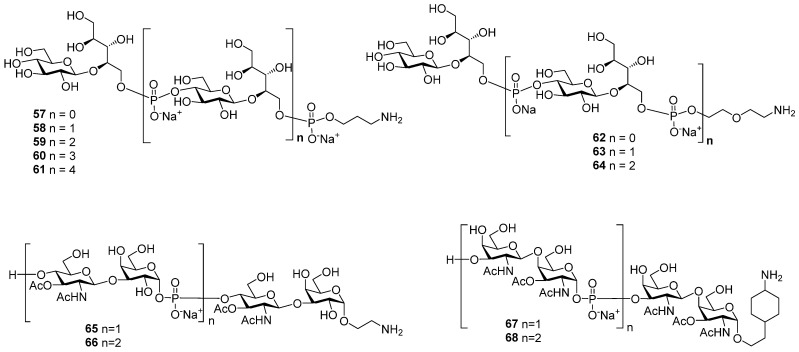
Synthetic oligomers **57**–**61** [94] and **62**–**64** related to *Hia* capsular phosphoglycans [96], oligomers **65** and **66** related to *Hic* capsular phosphoglycans, and oligomers **67** and **68** related to *Hif* capsular phosphoglycans [97].

Inspired by the success of the Quimi-Hib^®^ vaccine, which comprises synthetic oligomeric phosphoglycans structurally related to *Hib* capsular phosphoglycan, researchers synthesized [94,95] the series of oligomers **57**–**61** with one, two, three, four, and five repeating units of *Hia* capsular phosphoglycans and 3-aminopropyl linkers (Figure 6). In another recent study, the series of fragments of *Hia* capsular phosphoglycans **62**–**64** equipped with a **66** diethyleneglycol linker was obtained [96] (Figure 6). Syntheses of aminoalkyl glycosides related to the *Hic* phosphoglycan (compounds **65** and **66**) and related to the *Hif* phosphoglycan (compounds **67** and **68**) were performed by Oscarson et al. [97].

**Scheme 7:**
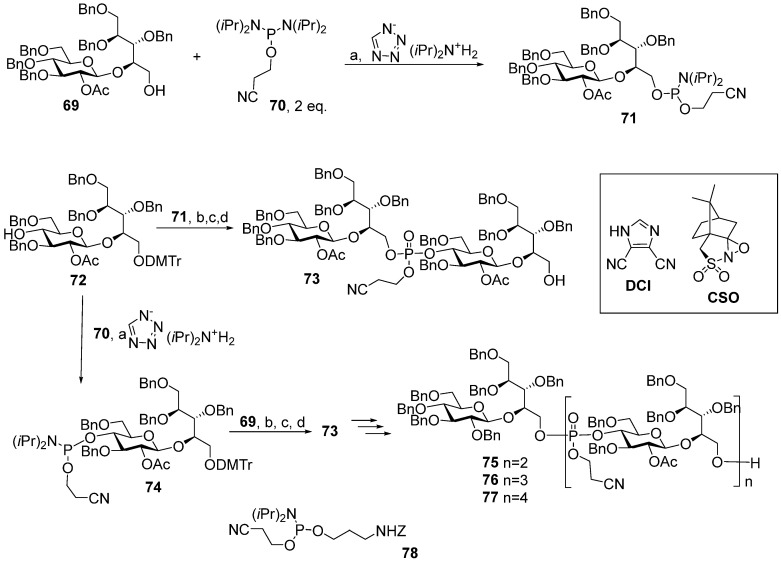
Key building blocks for the synthesis [94] of oligomers structurally related to *Hia* capsular phosphoglycans. Reagents and conditions: (**a**) CH_2_Cl_2_, r.t., 98% for compound **71** and 95% for compound **73**; (**b**) DCI, CH_2_Cl_2_, 4Å molecular sieves; (**c**) CSO; (**d**) 0.18M TCA in CH_2_Cl_2_, 79% over 3 steps.

**Scheme 8:**
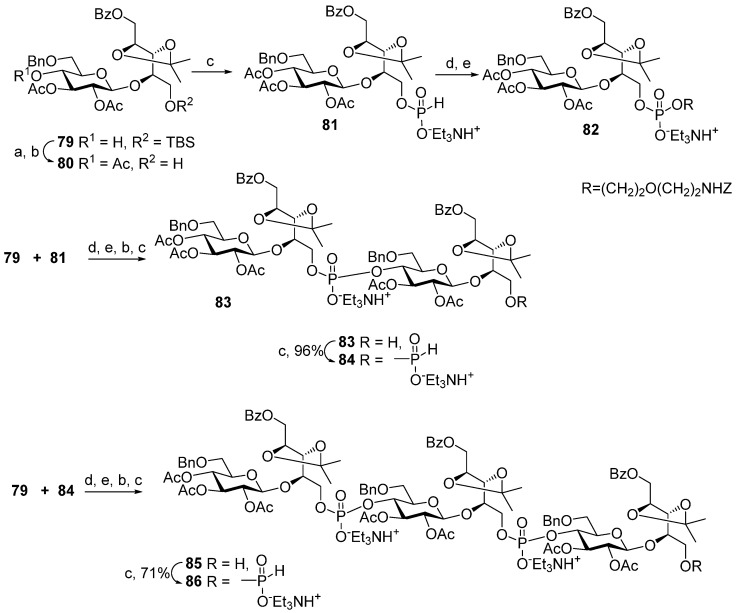
Key blocks for synthesis of oligomers structurally related to *Hia* phosphoglycans [96]. Reagents and conditions: (**a**) acetic anhydride, DMAP, Py; (**b**) HF (aq.), Py, 60 °C, 97% for two stages; (**c**) H_3_PO_3_, PivCl, Py, 74%; (**d**) ZNH(CH_2_)_2_O(CH_2_)_2_OH, PivCl, Py; (**e**) I_2_, Py/H_2_O.

In the two series of the spacer-armed oligomers **57**–**61** [94] and **62**–**64** [96] structurally related to *Hia* capsular phosphoglycans, the linker is connected to the glycan antigen via a phosphodiester bridge. Compounds in both series were prepared using a convergent synthetic strategy with iterative elongation of the linear structure starting from the non-reducing end, and finally, the spacer was added (Scheme 7 and Scheme 8). The major difference between the syntheses of these two series is the type of universal, selectively protected bifunctional synthon. In the preparation of oligomers **58**–**61** [94], a monomer block with a phosphoramide group at C-4 (Glc) was used, and for compounds **63**–**64** [96], the synthetic pathway involved the use of a non-phosphorus monomer.

For the choice of the optimum strategy for the assembly of oligomers **57**–**61** (Scheme 7) [95], two alternative ways were considered. The first strategy involved pseudo-disaccharide **69** with a free hydroxyl group in the ribitol residue, which was reacted with 2-cyanoethyl *N*,*N*,*N*′,*N*′-tetraisopropylphosphordiamidite (compound **70**) in the presence of diispropylammonium tetrazolide to obtain the selectively protected phosphoramidite **71** as a universal precursor with a 95% yield. The following condensation of phosphoramidite **71** and glycoside **72** with a free hydroxyl group at C-4 (Glc), oxidation of a phosphite into a phosphodiester, and detritylation created the conjugation-ready dimer **73** with a 73% yield over three steps.

Another way of preparing phosphotriester **73** (Scheme 7) employed phosphoramidite **74** with a pro-phosphodiester group located at C-4 (Glc). The sequential combination of phosphoramidite **74** with alcohol **69**, oxidation, and detritylation resulted in the formation of the phosphodiester block **73** with a 79% yield over three steps [94,95]. Therefore, both assembly strategies were equally effective. Starting with phosphoramidite **74**, protected pseudo-oligosaccharides **75**–**77** were obtained and then converted to ligands **58**–**61** by interaction with the pre-spacer phosphoramidite **78** and total deprotection. The authors pointed out that in comparison to the H-phosphonate approach, the phosphoramidite protocol was more efficient for the construction of longer phosphodiester-linked chains. Oligomers **57**–**61** were *N*-acylated with a di(*N*-hydroxysuccinimidyl) adipate linker, and the activated esters were conjugated to CRM197. The resulting conjugates contained 13–25 copies of the oligomeric antigen per CRM197 unit. Rats were immunized with three doses, which contained 2 μg of the glycan antigen, and the IgG antibodies were analyzed in the sera using ELISA. Conjugates of trimer **59** and pentamer **61** with HSA were used as coating antigens. All the conjugates were immunogenic and induced similar levels of IgG antibodies, which recognized immobilized synthetic antigens. The researchers noted that the immunogenicity data for the CRM197-based conjugates of oligomers **57**–**61** was independent of the length of the oligomeric antigen [95].

Compounds **62**–**64** [96] were prepared (Scheme 8) starting from the universal precursor pseudo-disaccharide **79**. The acetylation of compound **79** resulted in acetate **80**, which was phosphitylated to obtain key monomeric H-phosphonate **81** with a 97% yield. The condensation of H-phosphonate **81** with a pre-spacer *N*-carbamoyl aminopropanol, as shown in Scheme 8, created phosphodiester **82** (yield: 34%), which, after the removal of the protective groups, was transformed into the spacer-armed target monomer **62** (Figure 6). To obtain dimer **83**, alcohol **79** was condensed with H-phosphonate **81** with a 77% yield and then oxidized. Phosphodiester **83** was, in turn, converted into H-phosphonate **84**. The interaction of H-phosphonate **84** with alcohol **79** produced phosphodiester **85**, which was converted into H-phosphonate **86**. Spacer-armed derivatives were obtained by the interaction of H-phosphonates **84** and **86** with *N*-carbamoyl aminopropanol to obtain, after total deprotection, the spacer-armed dimer **63** and trimer **64**, respectively (Figure 6). It is interesting to note that the efficiency of condensation with N-protected aminopropanol increased in the series **81** > **84** > **86** with the rise in the number of phosphodiester fragments present in these H-phosphonates, whereas numerous experimental data suggest the opposite [39].

In 2001, Oscarson et al. synthesized the amino spacer-armed oligomeric fragments of *Hic* (compounds **65** and **66**) and *Hif* (compounds **67** and **68**) phosphoglycans [97] (Figure 6), in which the repeating disaccharide units are linked via a phosphodiester bridge. Similar to the block syntheses of *Hib* and *Hia* fragments described above, the establishment of a phosphodiester linkage between selectively protected oligosaccharide blocks was used for the chain elongation. However, unlike *Hib* and *Hia*, in *Hic* and *Hif* capsular phosphoglycans, the anomeric carbons in hexoses are involved in the formation of a phosphodiester bridge. Therefore, the development of a synthetic strategy for the preparation of *Hic* and *Hif* fragments has to be developed with respect to the possibility of the formation of an unwanted anomer. At the step of chain assembly, it can be particularly difficult to provide stereocontrol in the formation of a C-1-O-P bond. Instability of the anomeric phosphodiester linkage also has to be considered, which makes it possible to use anomeric phosphodiesters as glycosyl donors in the glycosylation reaction. A convenient strategy for the preparation of the desired anomer includes the initial stereocontrolled glycosylation of a phosphodiester synthon, which provides an intermediate product with the target anomeric configuration, and the subsequent coupling of this compound in mild conditions for the prevention of anomerization. These conditions are met in the H-phosphonate protocol, as hexose hemiacetals retain the anomeric configuration, while they are transformed into H-phosphonates upon the action of triimidazolyl phosphine prepared in situ from PCl_3_ and imidazole in the presence of Et_3_N [39].

**Scheme 9:**
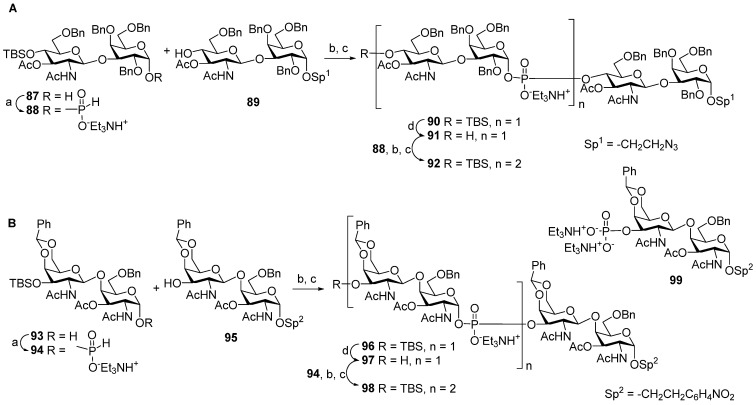
(**A**) Key blocks **87**–**92** for the synthesis of oligomers [97] **65** and **66** related to *Hic* phosphoglycans; (**B**) key blocks **93**–**98** for the synthesis of oligomers **67** and **68** related to *Hif* phosphoglycans. Reagents and conditions: (**a**) PivCl, Py; (**b**) PCl_3_, imidazole, Et_3_N, 85%; (**c**) I_2_, Py/H_2_O, -40 °C; (**d**) Et_3_N(HF)_3_.

For the preparation of the spacer-armed oligomers **65** and **66** (Figure 6) [97], which are structurally related to *Hic* phosphoglycans, hemiacetal **87** with an axial hydroxyl group at C-1 was converted into the key α-H-phosphonate 88 (yield: 89%), which was condensed with the selectively protected alcohol **89** and oxidized with I_2_ (Scheme 9A) to provide phosphodiester **90** (yield: 71%). The desilylation of phosphodiester **90** produced alcohol **91**, which was readily condensed with α-H-phosphonate **88**, and the phosphite group was oxidized to produce phosphate **92** in a moderate yield (36%). After total deprotection, the reduction of the azido group, and the N-acetylation of compounds **91** and **92**, the target spacer-armed phosphooligosaccharides **65** and **66** were obtained.

Analogously, the favorable α-configuration of the GalNAc hemiacetal residue in disaccharide **93** (Scheme 9B) paved the way to the preparation of the spacer-armed phosphooligosaccharides **67** and **68** related to *Hif* phosphoglycans [97]. Upon action with PCl_3_ and imidazole, hemiacetal **93** was transformed into α-H-phosphonate **94** with a retention of configuration (yield: 87%). The condensation of α-H-phosphonate **94** with disaccharide **95** and the subsequent oxidation resulted in the efficient formation of phosphodiester **96** (yield: 81%), which was desilylated to obtain alcohol **97**. Similar to the condensation of compounds **91** and **88**, the interaction of **97** and **94** was less efficient, and phosphodiester **98** was obtained with a 37% yield. Most likely, low yield in this reaction is associated with the high lability of the phosphodiester group in acceptors **91** and **97** in the conditions of oxidation with I_2_ [39]. The authors [97] observed the formation of a significant amount (up to 40%) of 3′-O-phosphate **99**, which indicates the lability of a glycosyl-O-phosphodiester tether under oxidation conditions. The low yields of attachment of the second glycosyl-phosphodiester residue for variants A and B, shown in Scheme 9, suggest that the stability of a phosphodiester bond is largely determined by the condensation and oxidation conditions and is less dependent on the structure of disaccharide residues. The target spacer-armed phosphooligosaccharides **67** and **68** were obtained after complete deblocking of the compounds and the reduction of the nitrophenyl residue into an aniline residue.

In view of the development of an anti-*Hia* vaccine, two aspects have to be addressed. Similar immunogenic properties of synthetic *Hia* antigens from monomer to pentamer can be connected with the low stability of phosphodiester linkages in aqueous solutions. In this case, the design of a vaccine may require a mimetic structure for the antigen. Also, it is advisable to use a phosphoramidite-based strategy for the preparation of phosphooligosaccharide antigens for industrial production, as the considered examples demonstrate the advantage of the phosphoramidite-based method over the H-phosphonate procedure. Meanwhile, anti-*Hic and anti-Hif* conjugate vaccines are not considered to be of high importance in contemporary glycoscience, as researchers have not referred to this topic for more than 20 years.

## 4. Synthesis of Phosphooligomers Related to Capsular Phosphoglycan of MenA

In 2010, a conjugate polysaccharide meningococcal monovalent vaccine, MenAfriVac^®^ (MenA-TT), was licensed [98,99]. In contrast to meningococcal polysaccharide vaccines, the conjugate preparation was found to be highly effective. For example, the results of mass vaccination campaigns in African meningitis belt countries that were carried out in 2010–2015 showed more than a 99% decrease in the incidence of *MenA*-associated meningitis [100]. Today, *MenA* phosphoglycan is a component of a number of polyvalent conjugate meningococcal vaccines, including Menactra^®^ (A-meningococcal component MenA-diphtheria toxoid), Menveo^®^ (A-meningococcal component MenA-CRM197), and Nimerix^®^ (A-meningococcal component MenA-TT) [14].

Polymer chains of *MenA* phosphoglycan are highly labile in aqueous media [63]. This intrinsic property of *MenA* phosphoglycan creates the need for the cold-chain transport of conjugate anti-*MenA* vaccines [101], which significantly increases the cost per dose and, in some cases, is an insurmountable obstacle to the use of this type of preparation [102]. Also, this feature imposes additional requirements on the production of both monovalent vaccines and polyvalent vaccines in a convenient liquid form. In this regard, considerable research efforts have been directed towards the synthesis of oligomeric antigens structurally related to *MenA* CPS fragments or corresponding mimetics with a view to preparing commercial conjugate vaccines. The use of anti-*MenA* conjugate preparations with a synthetic oligoside antigen, the structure of which fully corresponds to *MenA* CPS fragments, could allow the monitoring of the structural integrity of this preparation during storage and transportation, and the design of an antigen using ManNAc phosphate mimetics obtained by replacing the oxygen atom with the methylene group in the pyranose ring with (carba-analogs) or by replacing the anomeric oxygen atom with a methylene group (C-phosphonates) will make the antigen structure resistant to hydrolysis.

It is important to emphasize that in *MenA* bacterial phosphoglycans, ~80% of 3-OH groups and ~10% of 4-OH groups in ManNAc residues are acetylated [103]. A study of phosphoglycan structures present on the surface of a living *MenA* bacterial cell [104], which was conducted using high-resolution magic-angle spinning, showed the presence of acetyl substituents at 50–60% of 3-OH groups and 25–30% of 4-OH groups. The acetylation of *MenA* phosphoglycans is known to be an important antigenicity factor [105,106].

**Figure 7:**
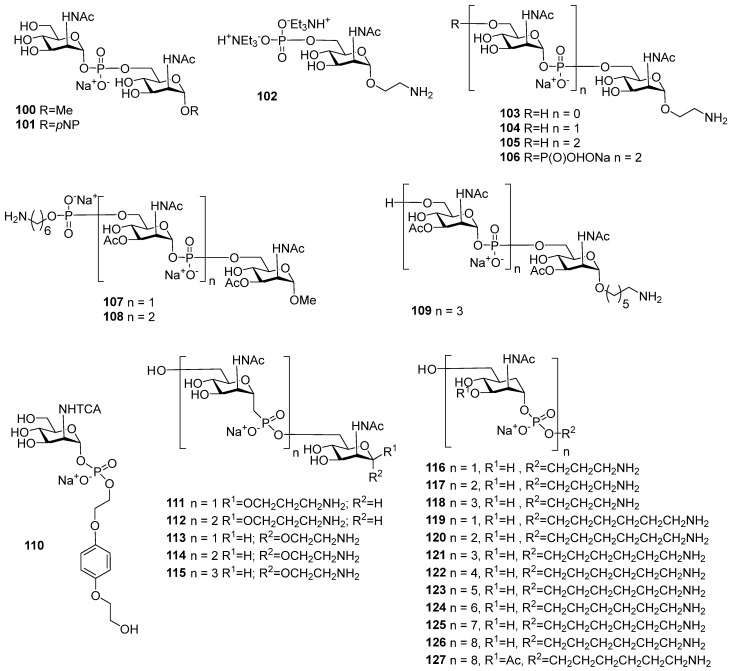
Synthetic oligosaccharides structurally related to *MenA* phosphoglycans.

Spacer-armed synthetic antigens structurally related to the *MenA* capsular phosphoglycan and the corresponding phosphono- and carba-analogs are attractive synthetic compounds considering their potential use as ligands in marketed meningococcal vaccines. Since the first time a phosphodiester bridge was arranged between two αManNAc residues (Figure 7, compounds **100** and **101**) in 1993 by the Shibaev group [107], a vast number of *MenA*-related mono-, di-, and oligosaccharides **102**–**109** (Figure 7) have been synthesized [108,109,110,111,112]. However, the preparation of αManNAc anomeric phosphodiesters remains a challenge, as these compounds comprise a highly labile linkage between C-1 and O-1 of αManNAc, which is, in addition, destabilized by the presence of the NHAc group at C-2 of αManNAc. As a result, researchers have focused significant efforts on the design of hydrolytically stable phosphono-mimetics (compounds **111**–**115**) [113,114,115] and carba-mimetics (compounds **116**–**127**) [116,117,118]. However, new challenges are emerging, which are associated with the establishment of C-C bonds and the stereoselective formation of new chiral centers. The majority of publications that describe the preparation of oligomeric antigens related to *MenA* phosphoglycans concern nonacetylated molecules, whereas bacterial *MenA* phosphoglycans comprise the acetyl groups at O-3/O-4 (Figure 6), which are important for the antigenic properties of the polymer [105,106]. Additionally, a number of synthetic *MenA*-related antigens were reported (compounds **107**–**109** and **127**), which are totally or partially acetylated at O-3 of αManNAc, with a view to bringing their antigenic properties closer to those of the bacterial antigen. Synthetic fragments of *MenA* phosphoglycans with 3,4-di-O-acetylated αGManNAc residues are not considered in this review.

**Figure 8:**
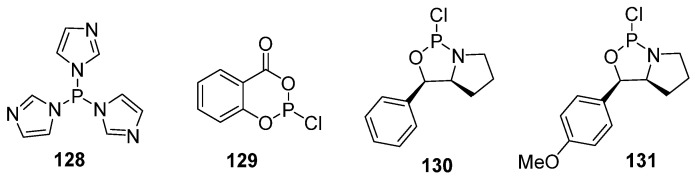
Reagents used for synthesis of H-phosphonates.

As the capsular MenA glycan is an αManNAc(1→(-PO_3_)→6) polymer, the H-phosphonate method is applicable, provided that 1-OH has the axial orientation in the selectively protected H-phosphonate ManNAc or 2-deoxy-2-azido mannose precursor, as described above for oligomers related to *Hic* and *Hif* phosphoglycans (Scheme 9). A number of convenient and efficient procedures have been developed for the preparation of this type of compound. The common protocol suggests phosphitylation with tri(1-imidazolyl) phosphine (Figure 8, compound **128**), which is obtained in situ by the interaction of PCl_3_ and imidazole in the presence of Et_3_N and salicylchlorophosphite **129** (2-chloro-4H-1,3,2-benzodioxaphosphin-4-one; Figure 8). Alternatively, within a phosphoramidite protocol, chloroanhydrides **130** and **131** (Figure 8) were especially designed for automated synthesis to produce stable phosphoramidites as synthons of α-mannosamine phosphodiesters [108].

For the PCl_3_/imidazole protocol, the key prerequisite for the preparation of ManNAc or 2-deoxy-2-azido mannose α-H-phosphonates is the α-configuration of the 1-OH group in the corresponding selectively protected hemiacetal. This feature imposes certain restrictions on the use of the H-phosphonate protocol. Hemiacetal **132** (Table 1) with a 5:1 ratio of α/β anomers was quantitatively converted into the 5:1 mixture of α- and β-isomeric H-phosphonate **133** upon the action of PCl_3_ and imidazole in acetonitrile at 0 °C, followed by hydrolysis with an aqueous solution of Et_3_NH∙HCO_3_ at 20 °C (Table 1, entry 1) [107]. In a similar way, phosphitylation of the mixture of hemiacetal **134** with a 3.5:1 ratio of α/β anomers in analogous conditions proceeded with the retention of the configuration of the anomeric center and resulted in the formation of the mixture of H-phosphonate **135**, with the α:β ratio 3.5:1 and an 88% yield (Table 1, entry 2) [110]. In similar conditions, the interaction of the α-anomer of hemiacetal **136** (Table 1, entry 3) [111] afforded α-H-phosphonate **137** with a 97% yield, and hemiacetal **138** with a dibenzyl phosphate group at C-6 was converted into α-H-phosphonate **139** (Table 1, entry 4) [111]. The phosphitylation of hemiacetal **140** (Table 1) with an α-configuration of the anomeric center upon the action of (PhO)_2_P(O)H in Py, followed by hydrolysis, resulted in a mixture of α- and β-isomer **141** in a ratio of 93:3 (Table 1, entry 5) [112]. Similarly, the phosphitylation of α-hemiacetal **142** in these conditions formed α-H-phosphonate **143** with a 78% yield (Table 1, entry 6) [109]. Anomeric mixtures of hemiacetals, which contain considerable quantities of β-isomers, can be efficiently converted into α-H-phosphonates with chlorophosphite **129** (Figure 8) [110,119]. For example, the phosphitylation of the anomeric mixture **144**, with a ratio of α- and β-anomers of 4.5:1, was transformed into α-H-phosphonate **145** by the action of chlorophosphite **129** in a mixture of dioxane and triethylamine with an 88% yield (Table 1, entry 8) [110]. Similarly, in these conditions, hemiacetals **142** and **146** were converted into the corresponding α-H-phosphonates **143** and **147** at 91 and 90% yields, respectively (Table 1, entries 7 and 9) [110,119]. Unlike H-phosphonates, phosphoramidites are rarely used as synthetic blocks for the establishment of phosphodiester bonds between α-ManNAc residues because of their utmost lability. Two successful examples of ManNAc phosphoramidites are compounds **149** and **150**, which are stabilized by rigid oxazaphospholidine substituents. These compounds were obtained from the anomeric mixture of hemiacetal **148** by the action of tricyclic chlorophosphoroamidites **130** and **131** at yields of 36 and 39% (Table 1, entries 10 and 11), respectively [108]. The generation of α-H-phosphonates and the anomerization of the α/β mixtures of hemiacetals or the stereoselective cleavage of the unwanted β-isomer in the presence of H_3_PO_3_ [107] or AgOTf [110] were ineffective.

**Scheme 10:**
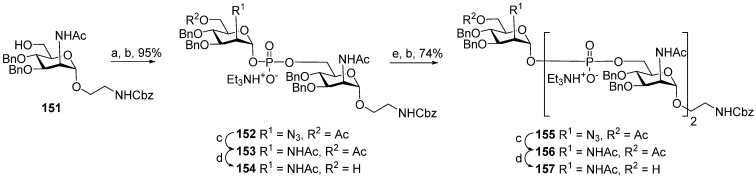
Key building blocks for the synthesis of oligomers **103**, **104**, and **105** related to *MenA* phosphoglycans [112]. Reagents and conditions: (**a**) **141** (0.8 eq.), PivCl (2.2 eq.), 23 °C, 30 min. Py; (**b**) I_2_ (2.2 eq.), Py:H_2_O 95:1, 0 °C, 30 min, 95% yield over 2 steps; (**c**) NiCl_2_∙6H_2_O (4.8 eq.), NaBH_4_ (8.0 eq.), methanol, 0 °C → 10 °C, 30 min; (**d**) NaOMe/MeOH; (**e**) **141** (1 eq.), PivCl (1.5 eq.), Py, 23 °C, 5 min.

The majority of syntheses of the spacer-armed oligomers related to *MenA* phosphoglycans (Figure 7) were performed using a straightforward and efficient protocol suggested by Van Boom [120], which constitutes the condensation of H-phosphonates with alcohols, with the subsequent oxidation of phosphites into phosphodiesters. In the first step, the dissolved alcohol is activated by the addition of sterically hindered chloroanhydride, e.g., PivCl, followed by the addition of H-phosphonate in Py and stirring for 5–30 min. In the second step, the intermediate products are subjected to oxidation with a 0.5 M solution of I_2_ in a Py:H_2_O mixture within the temperature interval from −40 °C to 0 °C for 30 min. The first syntheses of the non-acetylated fragments of *MenA* phosphoglycans as methyl glycoside **100** and nitrophenyl ether **101** were accomplished by the Shibaev group [107] using the H-phosphonate protocol (Figure 7). Then, monomers **102** and **103**, dimer **104**, and trimer **105** (Figure 7), related to *MenA* phosphoglycans and equipped with a spacer carrying a primary amino group for conjugation with protein carriers, were synthesized by Pozsgay et al. [112]. The condensation of H-phosphonate **141** with alcohol **151**, with subsequent oxidation, resulted in the formation of phosphodiester **152** with a yield of 95% over two steps (Scheme 10). The successive reduction of an azido group into the amino group and N-acetylation yielded mannosaminyl phosphodiester **153**, which was converted into alcohol **154** by chemoselective 6-O′-deacetylation. The condensation of H-phosphonate **141** and alcohol **154** was less effective (a yield of 74% over two steps) in connection with the destruction of the phosphodiester bond in the reaction conditions. The authors noted that attempts at further elongation of the chain were unsuccessful. Phosphoester **102** and phosphodiesters **103**, **104**, and **105** were converted into conjugates with HSA. The antigenic properties of the conjugates were confirmed by a double immunodiffusion assay with the *MenA* phosphoglycan as a positive reference and chemically modified HSA as a negative reference, and anti-*MenA* horse serum [112].

**Scheme 11:**
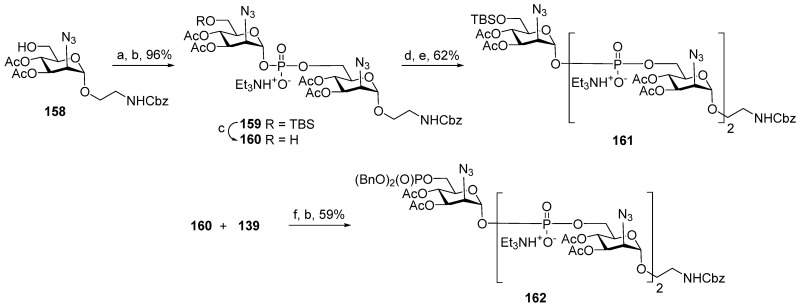
Key building blocks for synthesis of spacer-armed antigens **105** and **106** related to MenA phosphoglycans [111]. Reagents and conditions: (**a**) **137** (1.3 eq.), PivCl (3.8 eq.), Py, r.t., 60 min; (**b**) I_2_ (1.2 eq.), Py:H_2_O 49:1, −40 °C → 0 °C, 30 min, 96% yield over 2 steps; (**c**) Et_3_N∙3HF (5 eq.), THF, 91% yield; (**d**) **137** (1.3 eq.), PivCl (2.5 eq.), r.t., 120 min. Py; (**e**) I_2_ (1.2 eq.), Py:H_2_O 49:1, −40 °C → −10 °C., 62% yield over 2 steps; (**f**) PivCl, Py.

In 2005, the non-acetylated spacer-armed *MenA* phosphoglycan-related oligomer **105** (Figure 7), which comprises three ManNAc residues connected by phosphodiester bridges and trimer **106** (Figure 7), composed of three non-acetylated *MenA* repeating units, was synthesized by the Oscarson group [111]. In contrast to the abovementioned synthetic strategy designed by the Pozsgay group, which suggested the reduction of the azido group and N-acetylation after each chain elongation step (Scheme 10), 2-azido glycoside **158** was used as a starting unit for further chain elongation, and 2-azido H-phosphonate **137** (Table 1, entry 3) was applied as a repeating unit synthon without intermediate N_3_→NHAc transformation (Scheme 11). The condensation of H-phosphonate **137** and acceptor **158** with a free hydroxyl group at C-6, followed by oxidation, resulted in the efficient formation of phosphodiester **159** (yield: 96%), which was then 6′-O-desilylated to create a new active site in acceptor **160**. However, the attachment of the next monomer unit **137** to phosphodiester **160** was less effective and, after oxidation, formed the corresponding phosphodiester **161** with a 62% yield. With a view to synthesizing trimer **106** with a phosphoester substituent at C-6, acceptor **160** was reacted with phosphodiester **139** (Table 1, entry 4) and a dibenzylphosphate group at C-6 to obtain the trimeric precursor **162** with a 59% yield. A reduction in all azido groups, total N-acetylation, and deprotection in compounds **161** and **162** afforded the spacer-armed conjugation-ready antigens **105** and **106** [111].

**Scheme 12:**
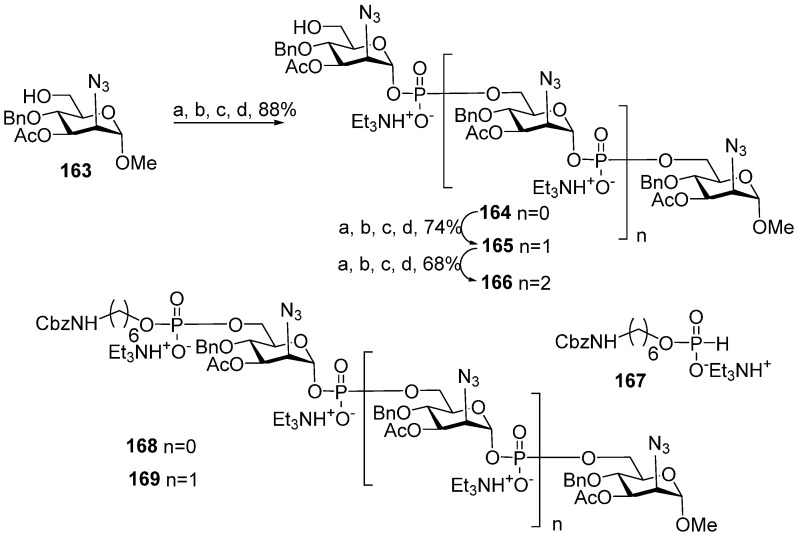
Key building blocks for synthesis of spacer-armed oligomers **107** and **108** [110] related to *MenA* capsular phosphoglycans. Reagents and conditions: (**a**) **145** (1.2 eq.), PivCl (1.8 eq.), Py, 20 °C, 30 min; (**b**) Et_3_N (4.8 eq.), −40 °C, Py:H_2_O 95:5; (**c**) −40 °C, I_2_ (2.4 eq.), 30 min; (**d**) 10 °C, 1% TFA in dichloromethane.

3-O-Acetylated dimer **107** and trimer **108** related to *MenA* capsular phosphoglycans were synthesized as methyl glycosides (Figure 7) [110]. The phosphitylation of acceptors **163**, **164**, and **165**, which comprised one, two, or three 2-deoxy-2-azido mannose residues with H-phosphonate **145**, followed by oxidation and detritylation, afforded phosphodiesters **164**, **165**, and **166** with 88%, 74%, and 68% yields, respectively. The alcohols obtained were subjected to phosphitylation with H-phosphonate **167** and subsequent oxidation (Scheme 12). The connection of H-phosphonate **167** to the 6-OH of the terminal monosaccharide was effective only for the shorter alcohols **164** and **165** (88% over two steps) and resulted in the preparation of protected di- and trimers **168** and **169** (Scheme 12). The transformation of azido groups into amino groups and the total N-acetylation and debenzylation of compounds **168** and **169** formed the spacer-armed dimer **107** and trimer **108**, which carry acetyl groups at O-3 of each ManNAc residue, as they do in bacterial *MenA* phosphoglycans. In the homologous series **164**–**166**, attempts to phosphitylate the longest alcohol **166** with H-phosphonate **145** in order to obtain a tetramer or with the pre-spacer H-phosphonate **167** were not successful, in contrast to compounds **164** and **165**.

**Scheme 13:**
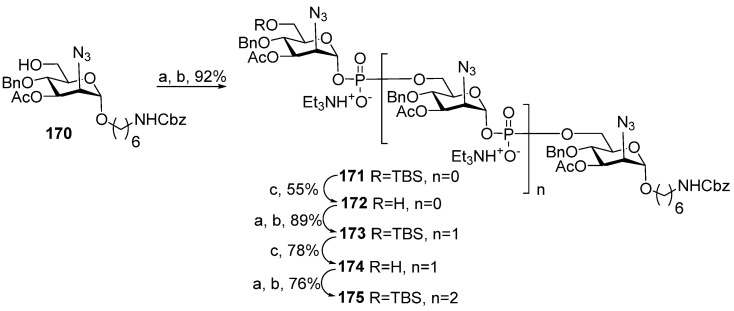
Key building blocks for the synthesis of oligomer **109** structurally related to *MenA* phosphoglycans [109]. Reagents and conditions: (**a**) **143** (2 eq.), PivCl (3 eq.), Py, 20 °C, 90 min; (**b**) −40 °C, solution of I_2_ (5 eq.) in Py/H_2_O 20:1, 150 min, −40 °C → −10 °C, 30 min; (**c**) Et_3_N∙3HF (5 eq.), THF.

In general, the examples of the synthesis of oligomers related to *MenA* capsular phosphoglycans discussed above are in line with the tendency outlined in an earlier review [39], which discussed the decrease in the efficiency of phosphitylation with H-phosphonates with the increase in the number of already formed phosphodiester bonds and suggested the lability of phosphodiester fragments in the conditions of the H-phosphonate protocol. However, in 2017, a group of Indian researchers synthesized an aminohexyl glycoside of oligomer **109** related to capsular phosphoglycans, which contained four 3-O-acetylated ManNAc residues, using a linear, synthetic strategy (Scheme 13) [109]. In a sequence of phosphitylation steps, H-phosphonate **143** was used as a universal monomer (Table 1, entry 6). The establishment of the first phosphodiester linkage between alcohol **170** and H-phosphonate **143** afforded compound **171** (yield: 92%), which was, in turn, desilylated to form acceptor **172**. The phosphitylation of alcohol **172** with H-phosphonate **143**, followed by oxidation, resulted in the formation of compound **173** with two phosphodiester bridges (yield: 89%). The desilylation of compound **173** formed acceptor **174**, which was phosphitylated with H-phosphonate **143** and, after oxidation, compound **175** with three phosphodiester bridges was obtained with a 76% yield.

After the transformation of azido groups into NHAc groups and the desilylation, debenzylation, and deprotection of the spacer amino group, the spacer-armed ligand **109** was conjugated to TT. The antigenic properties of oligomer **109** were evaluated in a competitive ELISA experiment. Both oligomer **109** and its conjugate with TT in the concentration range 12.5–400 μg glycan/mL were found to neutralize anti-MenA rabbit antiserum and inhibit the binding of antibodies to the bacterial *MenA* phosphoglycan used as a coating antigen. In comparison to the conjugate, oligomer **109** showed lower inhibition [109]. It can be concluded that the improvement of synthetic protocols made it possible to obtain oligomeric antigens related to *MenA* capsular phosphoglycans, which contain up to four ManNAc residues. However, for the efficient application of antigens of this type in immunodiagnostic tests and vaccine production, the hydrolytic lability issue has to be addressed.

## 5. Synthesis of Glycomimetics of MenA Capsular Phosphoglycans

Today, all anti-*MenA* conjugate vaccines include a lyophilized bacterial *MenA* component except for the fully liquid commercial vaccine preparation Menactra^®^. The presence of the *MenA* antigen in the dissolved form substantially shortens the shelf life for Menactra^®^ to 18 months at 2–8 °C compared to the 4-year shelf life of MenQuadfi^®^ and the 3-year shelf life of Menjugate^®^ and MenAfriVac^®^ in these conditions. With a view to preparing hydrolytically stable MenA antigens, a number of oligomeric analogs were designed, in which NHAc groups were replaced with trichloroacetamide groups as they are not likely to undergo transformation into oxazolines. Another type of mimetics is compounds with the isosteric replacement of one of the hemiacetal oxygens with a methylene group (phosphono- and carba-analogs).

**Figure 9:**
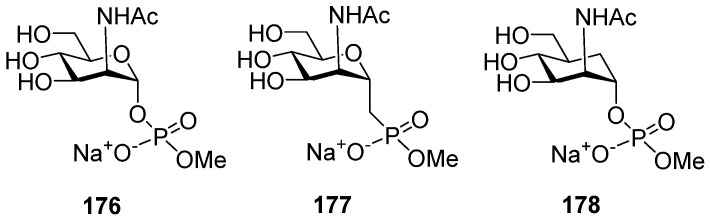
Sodium salt phosphodiesters of α-ManNAc (**176**) and its phosphono-analog (**177**) and carba-analog (**178**) as model compounds for comparative conformational analysis.

A comparative conformational analysis of a hexapyranose ring in 2-deoxy-2-acetamido mannohexapyranosyl phosphate **176** and its phosphono-analog **177** and carba-analog **178** (Figure 9) in a study of conformations in combination with NMR experiments showed that for compound **176**, 4C1 was almost the only populated conformer, whereas for phosphonate **177**, the proportion of pyranose ring conformers other than 4C1 was 4%, and for the carba-analog **178**, this proportion rose to 7% [121]. The most populated 4C1 conformer for compound **178** was confirmed by quantum mechanics and molecular dynamics calculations [122]. The molecular dynamics calculations performed for a decamer of a *MenA* capsular phosphoglycan repeating unit and its carba-analog showed that, despite a number of conformational and dynamic variations, the carba-mimetics of the fragments of *MenA* capsular phosphoglycans can be considered candidate antigens for the construction of anti-*MenA* vaccine preparations [123].

**Scheme 14:**
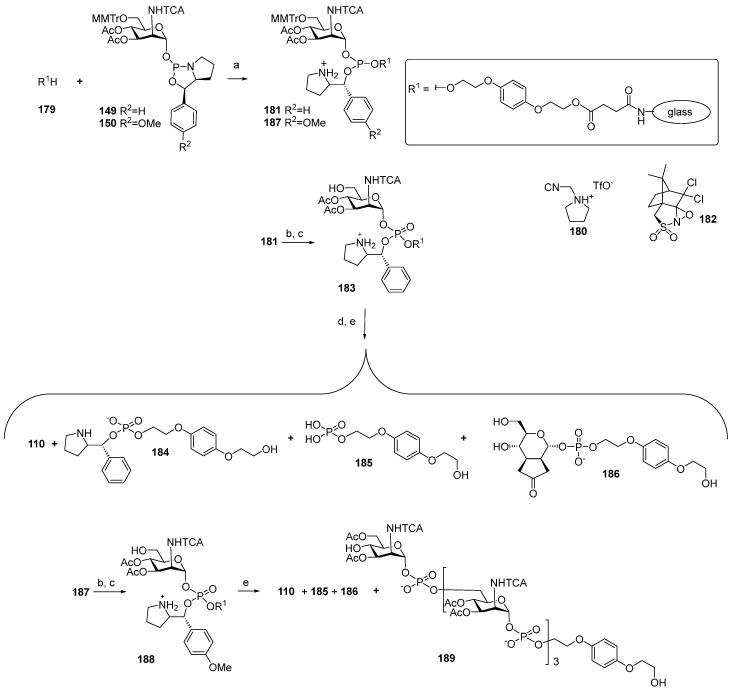
Key building blocks for the solid-phase synthesis of a monomer of *MenA* capsular phosphoglycans, **110**, in which the NHAc moiety has been substituted for trichloroacetamide [108]. Reagents and conditions: (**a**) **180**, r.t., acetonitrile; (**b**) **182**, acetonitrile; (**c**) 1% THF, CH_2_Cl_2_, TES, r.t., 1 min., 1 min.; (**d**) 2,6-lutidine, acetonitrile; (**e**) NaOMe/MeOH.

As mentioned previously, the axial NHAc group at C-2 of the ManNAc residue contributes largely to the lability of *MenA* capsular phosphoglycans and their fragments via neighboring group participation. One of the possible ways to circumvent this obstacle is the replacement [55] of the acetamide group at C-2 with the trichloroacetamide group, which is not likely to form oxazolines. To study the possibility of the preparation of the N-trichloroacetamide mimetics of ManNHAc, oxazaphospholidines **149** and **150** (Table 1) were obtained in a solid-phase synthesis. Bis(2-hydroxyethyl) hydroquinone **179** (Scheme 14) [108] was immobilized on a glass support and phosphitylated with phosphoramidites **149** and **150**. The phosphitylation of hydroquinone **179** with phosphoramidite **149** in the presence of 1-(cyanomethyl) pyrrolidinium trifluoromethanesulfonate (compound **180**) resulted in the formation of phosphite **181**, which carried a 2-phenylpyrrolydine residue as a result of the cycle opening. Phosphotriester **181** was subjected to mild oxidation with DCSO (compound **182**) and desilylated with TFA/TES to produce phosphotriester 183, with a free 6-OH group for the following elongation of the oligomeric chain. Finally, the cleavage of the 2-phenylpyrrolydine ether was fulfilled in the presence of a base, followed by deacetylation and removal of the solid support by the action of MeONa/MeOH.

However, researchers faced considerable difficulties at the step of the cleavage of the 2-phenylpyrrolydine ether. After the support was removed, analysis of the products showed that the use of DBU for the cleavage of the ether did not provide target phosphodiester **110**, and the identified products **184**–**186** did not contain a ManNAc residue. When the weaker bases of Et_3_N or 2,6-lutidine were used as basic catalysts, the target product **110** was obtained with a moderate yield (17%) and low conversion.

In order to replace the step of the basic hydrolysis of the O(P)-protective group for acidic hydrolysis, phosphoramidite **150** with a p-MeO-Ph residue in the place of the Ph residue, as in compound **149**, was used. Phosphoramidite **150** was attached to a solid support, and phosphite **187** was oxidized with DCSO (compound **182**) to yield phosphotriester **188**, followed by the simultaneous acid hydrolysis of the trityl group and removal of the 2-(p-methoxybenzyl) pyrrolidine moiety. However, after deacetylation and removal of the solid support with 50 mM NaOMe/MeOH, the unwanted products **186** and **187** were detected. Without taking into account inefficient deprotection, this method provided the preparation of tetramer **189**, which was O-acetylated and N-trichloroacetylated. The authors noted that the developed method in the current state is not suitable for the synthesis of oligomers [108].

**Scheme 15:**
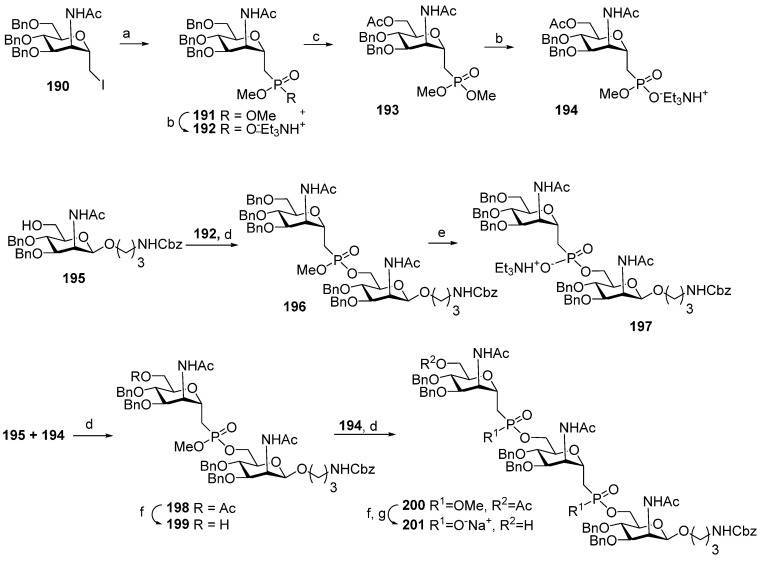
Key blocks for the synthesis of spacer-armed phosphono-analogs of *MenA* phosphoglycans, **111** and **112** (Figure 6) [113,115]. Reagents and conditions: (**a**) P(OMe)_3_, 100 °C, vacuum; (**b**) Et_3_N, PhSH, THF, r.t., 62%; (**c**) ZnCl_2_, Ac_2_O:AcOH 2:1, r.t., 16 h, 92%; (**d**) Ph_3_P, DIAD, THF, 0 °C, 24 h, yield 97% for **197**, 90% for **198**, 83% for 200; (**e**) Et_3_N, PhSH, toluene, 110 °C, 36 h, Amberlite IR120 (Na^+^) 95%; (**f**) NaOMe/MeOH; (**g**) DBU, PhSH, THF, r.t., 8 h, Amberlite IR120 (Na^+^), 95%.

In 2005, the preparation of the first isosteric hydrolysis-resistant phosphono-analog **111** structurally related to *MenA* phosphoglycans was published by Lay et al. [115]. The interaction of iodide **190**, in which the iodomethylene group is arranged axially, with trimethylphosphite (Scheme 15) afforded phosphonodiester **191**, which was further converted to ester **192** by the action of triethylamine and thiophenol.

Similarly, 6-O-acetylated phosphonodiester **193** was converted to the corresponding ester **194** by partial hydrolysis. Mitsunobu reaction conditions were used for the condensation of ester **192** with the selectively protected ManNAc **195**, which formed phosphonodiester **196** (yield: 97%) [113]. Partial hydrolysis of phosphonodiester **196** into phosphonoester **197** was also efficient. 6′-O-Acetylated phosphonodiester **198** was synthesized by the condensation of **195** with phosphonoester **194**, with a 90% yield. By deacetylation, phosphonodiester 198 was converted into alcohol **199** and condensed with phosphonoester **194** to form compound **200** with two phosphonodiester bridges and an 83% yield. Chemoselective hydrolysis of the methyl phosphonate moieties in compound **200** formed phosphonoester **201**. Total deprotection of phosphonoester **197** with two ManNAc residues and **201** with three ManNAc residues resulted in phosphono-analogs **111** and **112** (Figure 6) [113].

**Scheme 16:**
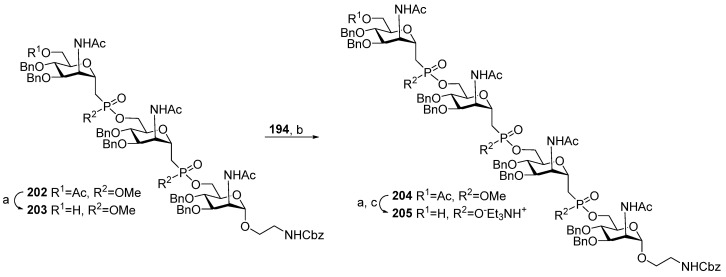
Key blocks for the synthesis of phospho-analog **115** (Figure 6) related to *MenA* phosphoglycans [114]. Reagents and conditions: (**a**) 1 M KOH/MeOH, 84%; (**b**) (p-ClC_6_H_4_)_3_P, DIAD, THF, 0 °C, 30 min, 87%; (**c**) DBU, PhSH, acetonitrile, r.t., 75%.

A similar strategy in combination with Mitsunobu reaction modification, where Ph_3_P is replaced with tris(4-chlorophenyl) phosphine, was used for the synthesis of the series of phosphono-analogs **113**–**115** of *MenA* phosphoglycans (Scheme 16) [114]. The 6″-O-deacetylation of phosphonodiester **202** resulted in alcohol **203**, which was then reacted with universal monosaccharide block **194** in modified Mitsunobu conditions to obtain the phosphono-analog **204** of *MenA* phosphoglycans with three phosphonodiester-bridged fragments (yield: 87%). Partial hydrolysis of diester moieties afforded compound **205** (yield: 75%), and after total deprotection, phosphono-analog **115** was obtained with three pseudo-ManNAc residues.

The antigenic properties of ligands **111** and **112** [113] were studied in competitive ELISA experiments with anti-*MenA* human antisera. Native *MenA* phosphoglycans were used as a coating antigen and positive control, and *MenY* phosphoglycans were used as a negative control. For both ligands, the EC50 was about 10^−3^ mg/mL, which is three orders higher than the EC50 of 6.6 × 10^−6^ mg/mL for *MenA* phosphoglycans.

**Figure 10:**
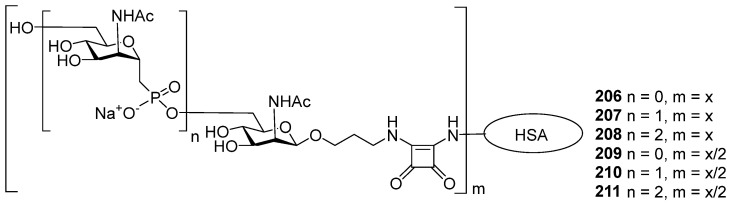
Conjugates of amino-spacered C-phosphono analogs of *MenA* capsular phosphoglycans [124].

Aminopropyl glycosides **111** and **112** and 3-aminopropyl β-D-ManNAc were transformed into conjugates with HSA using the squarate procedure [124] (Figure 10). One series consisted of conjugates **206**–**208** (Figure 10) with the maximum saccharide/protein molar ratio, and another series of conjugates was composed of compounds **209**–**211** (Figure 10) with the saccharide/protein molar ratio being half of the value achieved for conjugates **206**–**208**. Competitive ELISA experiments with the mouse polyclonal anti-*MenA* antisera and *MenA* capsular phosphoglycans as a coating antigen showed that at an inhibitor concentration of 1 mg/mL, inhibition using the fully loaded conjugate **206** was 55%. For conjugates **207** and **208**, it reached 65%, whereas *MenA* capsular phosphoglycans provided 100% inhibition. Inhibition with the half-loaded conjugates **209**–**211** was 5–15% lower than for the fully loaded conjugates with the same antigen type [124]. Conjugates **206**–**211** were used for the immunization of mice at a dose of 2 μg/mouse and efficiently evoked IgG antibodies, which is a reliable marker of the induction of thymus-dependent immune responses. Quantitative elucidation of the level of anti-*MenA* IgG antibodies developed against conjugates **206**–**211** showed that immunization with the half-loaded conjugates **209**–**211** was more efficient compared to the fully loaded conjugates **206**–**208**. It is important to note that the level of induced anti-*MenA* antibodies was similar for the half-loaded conjugates **209**–**211**, regardless of the length of the pseudo-oligosaccharide antigen. The authors concluded that this result indicated the antibodies’ recognition of the ManNAc epitope [124].

In contrast to C-phosphono mimetics, in which the methylene group stands in the place of anomeric oxygen, in carba-analogs, the decrease in electrophilicity of the carbonyl carbon, along with the increase in the stability of the phosphodiester linkage, is achieved by the replacement of the ring hemiacetal oxygen of ManNAc with a methylene group [125].

**Scheme 17:**
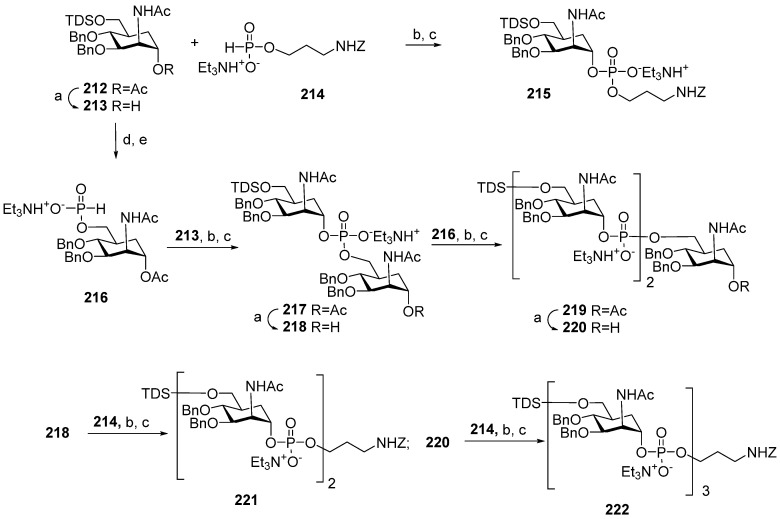
Key blocks for preparation of spacer-armed carba-analogs of oligomers **116**–**118** related to *MenA* CPS [118]. Reagents and conditions: (**a**) NaOMe/MeOH, r.t., 4 h, yield of compound **213**—84%, **218**—87%, **220**—70%; (**b**) Py, PivCl, r.t., 45 min; (**c**) I_2_, Py -H_2_O 19:1, r.t., 15 min, yield for compound **215**—81%, **217**—82%, **219**—81%, **221**—85%, **222**—57%; (**d**) Bu_4_NF, THF, r.t., 3 h; (**e**)–**129**, CH_3_CN-Py 3:1, r.t., 45 min, yield 82%.

Synthesis of the series of carba-analogs **116**–**118** (Figure 7) of aminopropyl glycosides of a monomer, a dimer, and a trimer of *MenA* capsular phosphoglycans, and the advanced series of carba-analogs **119**–**126**, from monomers to octamers, as aminohexyl glycosides, was performed by the Lay group [116,117,118]. Carba-analogs **116**–**118** [118] were obtained by the sequential elongation of a pseudo-oligosaccharide chain, starting from the spacer-equipped monomer (Scheme 17). For the preparation of monomer **116**, the universal orthogonally protected precursor **212** was transformed into alcohol **213**, which was phosphitylated with H-phosphonate **214** and oxidized to obtain the spacer-armed phosphodiester **215** with an 81% yield.

For the preparation of oligomers **117** and **118**, the universal precursor **212** was desilylated and converted into H-phosphonate **216**, which was used as a universal monomer block for chain elongation. The interaction of H-phosphonate **216** with alcohol **213** and the subsequent oxidation formed phosphodiester **217** (yield: 82%), which was deacetylated to obtain alcohol **218**. The condensation of alcohol **218** with H-phosphonate **216** resulted in pseudo-trisaccharide **219** (yield: 81%), which was deacetylated to produce alcohol **220**. Finally, pre-spacer **214** was introduced into alcohols **218** and **220** to obtain the spacer-armed, protected dimers **221** and **222** with yields of 85% and 57%, respectively. The high efficiency of the phosphitylation of alcohol **218**, which already includes a phosphodiester bridge with H-phosphonates **214** and **216**, evidences the increase in stability of the phosphodiester linkage in protected carba-analogs compared to phosphodiester-linked oligosaccharides. However, the phosphitylation of **220**, which already comprised two phosphodiester moieties, was less efficient, indicating the limitations of the application of the H-phosphonate procedure to the linear synthesis of longer oligomers. The target carba-analogs **116**–**118** of monomers, dimers, and trimers related to *MenA* phosphoglycans were obtained after the total deprotection of compounds **215**, **221**, and **222** [118].

**Scheme 18:**
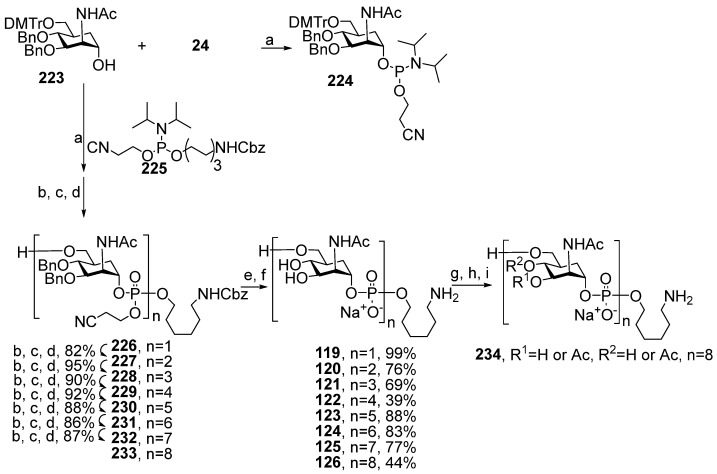
Key blocks for synthesis of spacer-armed carba-analogs **119**–**126** related to *MenA* capsular phosphoglycans [116]. Reagents and conditions: (**a**) DIPEA, CH_2_Cl_2_, r.t., 94%; (**b**) DCI, MeCN; (**c**) DCSO, MeCN; (**d**) TCA, CH_2_Cl_2_, H_2_O, 94% over 3 steps; (**e**) NH_4_OH, H_2_O, dioxane; (**f**) H_2_, Pd, H_2_O, AcOH, 44%; (**g**) (Boc)_2_O, NaHCO_3_, r.t. 16 h; (**h**) Ac_2_O/imidazole, 9 d.; (**i**) TFA, r.t., 1 h.

For the synthesis of the advanced series of carba-analogs **119**–**126**, from monomers to octamers, the Lay group used the strategy of a step-by-step chain extension using the spacer-equipped monomer as a starting compound and phosphitylation with a selectively protected monomeric phosphoramidite as the key step (Scheme 18) [116]. Alcohol **223** was converted into the universal phosphoramidite block **224** under the action of chlorophosphoroamidite **24**. The interaction of alcohol **223** with the phosphoramidite pre-spacer **225** in the presence of DCI, followed by oxidation with DCSO and detritylation, yielded phosphotriester **226** with a free hydroxyl group for further phosphitylation, which was used as a starting compound for chain elongation. The sequential execution of phosphitylation with the universal monomer block **224**, oxidation, and detritylation afforded the protected compounds **227**–**233** with excellent yields. After total deblocking, the spacer-armed carba-analogs **119**–**126**, structurally related to MenA capsular phosphoglycans, were obtained. For better resemblance of the octamer antigen to the natural structure, octamer **126** was N-Boc-protected, subjected to random monoacetylation, and N-deblocked, and the mixture of oligoacetates **234** was obtained, which contained a certain amount of oligomer **127** related to MenA capsular phosphoglycans [116].

The stability of compound **126** was studied using an accelerated stability test and fragments of natural and deacetylated *MenA* phosphoglycans, with avDP15 as a reference compound. During four weeks of keeping these samples in buffered 5 mM sodium acetate at pH 7 and 37 °C, a drastic decrease in chain length for the deacetylated bacterial oligosaccharide was observed, and the natural sample with acetyl groups was subjected to partial depolymerization, and for compound **126**, no trace of decomposition was detected [116].

**Figure 11:**
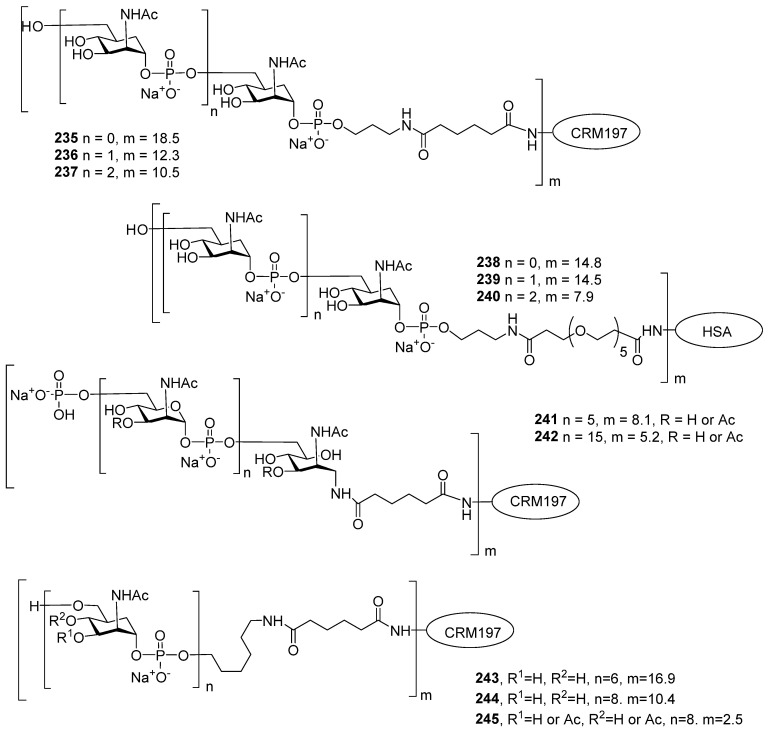
Neoglycoconjugates of mono- and oligomeric carba-analogs and partially fragmented *MenA* CPS with protein carrier CRM197 and HSA [116].

Competitive ELISA experiments with polyclonal immune mouse anti-*MenA* sera and *MenA* capsular phosphoglycans as a coating antigen were used for the assessment of the antigenic properties of the mono-, di-, and trimeric pseudo-oligosaccharides **116**–**118** [118]. *MenA* capsular phosphoglycans and their derivatives *MenA* avDP15 and *MenA* avDP3 were used as positive controls. The highest inhibition rate was found for *MenA* capsular phosphoglycans and *MenA* avDP15, with an IC50 of 5.15 × 10^−6^ and 4.3 × 10^−3^ mM, respectively. For the carba-dimer **117**, the IC50 was about 0.16–0.091 mM, which is less than the IC50 (4.3 × 10^−2^) found for *MenA* avDP3. For the carba-monomer **116** and carba-trimer **118**, only 30% inhibition was achieved [118]. The synthetic carba-analogs **116**–**118** were converted into conjugates with CRM197 (compounds **235**–**237**, Figure 11) and HSA (compounds **238**–**240**, Figure 11). Mice were immunized three times with conjugates **235**–**237**, **241**, and **242**, which comprised *MenA* avDP5 and *MenA* avDP15 antigens, respectively, at a dose of 2 μg of antigen per mouse. The induced antibodies were analyzed in ELISA experiments using the HSA-based conjugates **238**–**240** as coating antigens and *MenA* capsular phosphoglycans. The immunogenicity of conjugates **235**, **236**, and **237** was evaluated using the HSA-conjugated coating antigens **238**, **239**, and **240**, respectively. In this experiment, the immunogenicity of conjugate **238**, which comprised the monomeric antigen **116**, was somewhat lower compared to conjugates **239** and **240** with dimeric and trimeric antigens, respectively. In ELISA experiments with *MenA* capsular phosphoglycans as a coating antigen, it was found that only conjugate **240** with a trimeric antigen evoked antibodies specific to the natural phosphoglycans, and conjugates **238** and **239** did not induce anti-*MenA* immunity. However, the level of IgG antibodies induced by immunization with **240** was 2–3 orders lower than that induced by conjugates **241** and **242**, which contained fragments of natural phosphoglycans with intact acetyl substituents. These data are in line with the results of an in vitro bactericidal assay, which showed the intensity of the complement-mediated lysis of *N. meningitidis* [118].

The carbocyclic hexamer **124** and the non-acetylated and acetylated octamers **126** and **234** were conjugated to CRM197 to form compounds **243**, **244**, and **245**, respectively (Figure 11). Conjugates **243**–**245** were used for the immunization of mice, and another group of mice was immunized with a conjugate composed of partially depolymerized *MenA* phosphoglycans as a positive control. ELISA analysis of IgG antibodies on plates coated with *MenA* capsular phosphoglycans showed that immunization with conjugate **245**, based on the acetylated carba-octamer, efficiently induced anti-*MenA* immunity. For this conjugate, the IgG titers were comparable to the titers registered for conjugate **242** based on fragmented bacterial antigens, whereas the titers measured in animals immunized with conjugates **243** and **244**, which carried non-acetylated synthetic antigens, were two orders lower [116].

The urgency of the development of anti-*MenA* conjugate vaccines is evidenced by the considerable efforts of several research groups aimed toward antigenic and non-hydrolyzable synthetic phosphooligosaccharides. However, the antigenicity studies of phosphono- and carba-mimetics show that these oligomers do not induce anti-*MenA* immunity and, therefore, are unable to prevent MenA infection. Another obstacle in the way of the design of the semisynthetic anti-MenA vaccine is the importance of acetyl groups at the O3/O4 of ManNAc for the protective properties of the vaccines. The introduction of these substituents increases production costs.

To date, synthetic *MenA* antigens cannot compete with multiple conjugate anti-*MenA* vaccine preparations with a bacterial antigen. However, the repeating unit of *MenA* phosphoglycans is a simple monosaccharide, and it is a suitable target for production on automated synthesis platforms. We expect that the development of a stable mimetic with high antigenicity, in combination with automated synthesis technology, will help create an affordable anti-*MenA* semisynthetic conjugate vaccine.

## 6. Synthesis of Phosphooligomers Related to MenX Capsular Phosphoglycans

High anti-*MenA* vaccination coverage in the African meningitis belt in 2010–2015 resulted in a significant reduction of *MenA*-associated invasive diseases [100,126]. At the same time, outbreaks of invasive diseases caused by *MenX* were registered in Sub-Saharan Africa [127,128]. The clinical and epidemiological characteristics of diseases associated with *MenX* are similar to those of *MenA*. Over 90% of cases are registered during the dry season, the median age of patients is 9.2 years, and the mortality rate is 11.9% [129]. As a result, the development of a conjugated anti-*MenX* vaccine became an urgent issue [130].

For the elucidation of the minimal immunogenic epitope of *MenX* capsular phosphoglycans, a bactericidal mAb, *MenX*.01, was obtained, which induced bactericidal killing at a physiological concentration of 1 μg/mL in the first step. The interaction of this antibody with fragments of the natural *MenX* capsular phosphoglycans of different lengths showed that the minimal immunogenic epitope comprises 5–6 repeating units [131].

**Figure 12:**
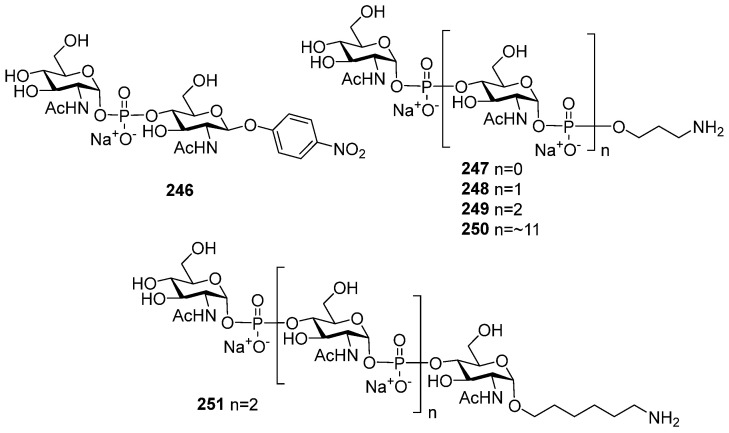
Synthetic phosphooligosaccharides **246**–**251** structurally related to *MenX* capsular phosphoglycans.

With a view to developing a conjugated anti-*MenX* glycovaccine with a fully synthetic glycan epitope, phosphodiester **246** [132] and the series of spacer-armed mono-, di-, and trimeric phosphoglycans **247**–**249** [129,133], were synthesized. In addition, a mixture of oligomeric phosphoglycan **250**, with an average length of 12 repeating units, was prepared using a combined chemo-enzymatic protocol, and pseudo-tetrasaccharide **251** was used as a glycoside with an aminohexyl spacer attached as an aglycon (Figure 12) [134].

The preparation of the synthetic oligosaccharide fragments of *MenX* capsular phosphoglycans was most commonly performed using an H-phosphonate procedure for the establishment of the phosphodiester linkage. In *MenX* phosphoglycans, the GlcNAc residue was incorporated in the polymer chain as an α-anomer. In synthetic fragments, the anomeric configuration of GlcNAc was determined with the configuration of the H-phosphonate, which, in turn, depends on the configuration of C-1 in the starting hemiacetal.

**Scheme 19:**
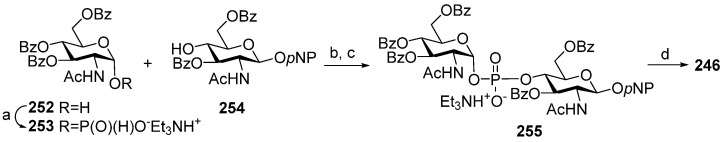
Key blocks for the synthesis of pseudo-disaccharide fragment **246** structurally related to *MenX* capsular phosphoglycans [132]. Reagents and conditions: (**a**) PCl_3_ (14 eq.), imidazole (4.3 eq.), Et_3_N (15 eq.), acetonitrile, 0 °C, 30 min, 95%; (**b**) PivCl (2.5 eq.), Py, 20 °C, 30 min. 72%; (**c**) Py:H_2_O 95:5; I_2_ (2.0 eq.), 20 °C, 10 min; (**d**) MeONa/MeOH, 71%.

In 1991, pseudo-disaccharide **246** was synthesized for the first time by the Shibaev group (Scheme 19) [132]. Hemiacetal **252** with the NHAc group at C-2 was transformed into α-H-phosphonate **253** with a 95% yield under the action of in situ generated tris(imidazol-1-yl) phosphine. The condensation of H-phosphonate **253** and alcohol **254**, followed by oxidation, afforded phosphodiester **255** (yield: 72%), which was deblocked to obtain pseudo-disaccharide **246** (Figure 12).

**Scheme 20:**
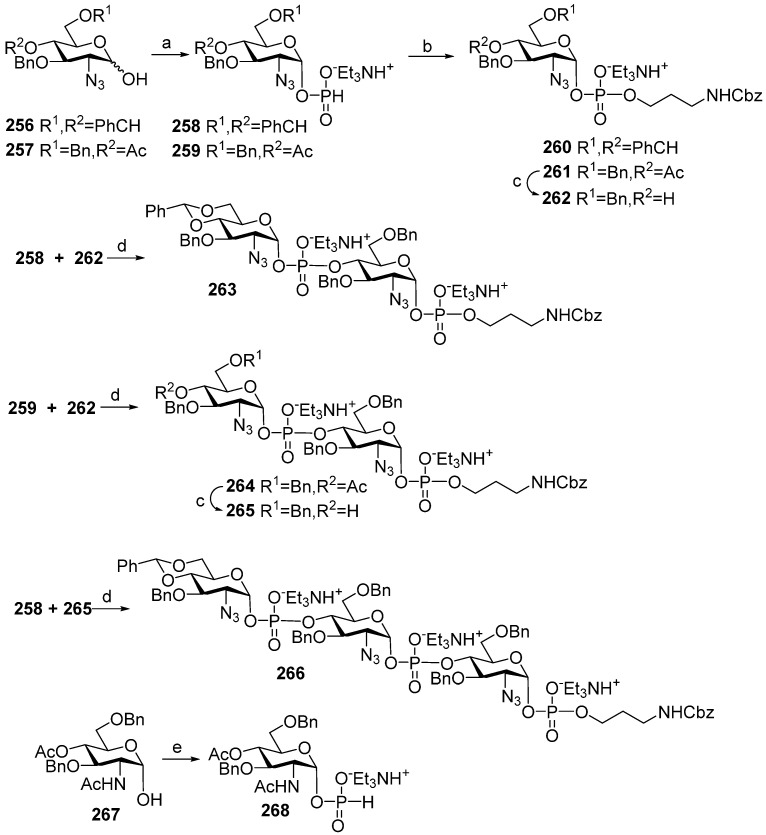
Key building blocks for the synthesis of pseudo-oligosaccharides **247**–**249** structurally related to *MenX* capsular phosphoglycans [129,133]. Reagents and conditions: (**a**) **129**, 2 M H_3_PO_3_/Py, 0 °C → 40 °C, 8–9 days, yield for **258**— 41% (+45% of starting material **256**), yield for **259**—52% (+38% of starting **257**); (**b**) HOCH_2_CH_2_CH_2_CH_2_NHCbz, PivCl (2.5 eq), Py, 45 min, followed by 0.5 M I2 in Py:H_2_O 19:1 (2.5 eq.), −40 °C, 10 min, yield over 2 steps for compound **260**—62%, for compound **261**—64%; (**c**) MeONa/MeOH, (**d**) PivCl (2.5 eq.), Py, 45 min, followed by 0.5 M I_2_ in Py:H_2_O 19:1 (2.5 eq.); 1 M triethylammonium bicarbonate buffer; (**e**) **129**, 1M triethylammonium bicarbonate buffer, batch 62% yield, microreactor 76% yield.

In 2013, the series of spacer-armed oligomers **247** –**250** was synthesized by the Lay group [133]. Unsuccessfully, 2-deoxy-2 azido hemiacetals **256** and **257**, intended for the preparation of the corresponding H-phosphonates **258** and **259**, were obtained as anomeric mixtures, thus challenging the preparation of H-phosphonates **258** and **259** as α-anomers. In order to provide the α-stereoselectivity of phosphonylation, the reaction of hemiacetals **256** and **257** with chlorophosphite **129** was conducted in the presence of phosphoric acid, which is known to destroy the β-anomer [39] (Scheme 20). The reaction took more than a week and afforded α-H-phosphonates **258** and **259** with 41% and 52% yields, respectively.

The phosphitylation of benzyl N-(3-hydroxypropyl) carbamate with benzylidenated H-phosphonate 258, followed by oxidation, resulted in phosphodiester **260** (yield: 62%), and the condensation of this alcohol with 4-O-acetylated H-phosphonate **259** formed phosphodiester **262** (yield: 64%), which was deacetylated to afford acceptor **262**. The condensation of acceptor **262** with 4,6-O-benzylidenated H-phosphonate **258** and 4-O-acetylated H-phosphonate **259** was conducted with a moderate yield of phosphodiester **263** (45%) and **264** (40%). After deacetylation, pseudo-disaccharide **264** was converted into alcohol **265**, which was reacted with 4,6-O-benzylidenated H-phosphonate **258** to afford trimer **266** with a 33% yield. The authors attribute the low efficiency of the phosphodiester linkage formation in trimer **266** to the presence of the azido group at C-2 of the hexapyranose ring. Total deprotection of compounds **260**, **263**, and **266** and transformation of the azido group into NHAc produced the target spacer-armed mono-, di-, and trimeric phosphodiesters **247**, **248**, and **249**, structurally related to MenX capsular phosphooligoglycan [133].

Unlike 2-deoxy-2-azido hemiacetals **256** and **257**, hemiacetal **267** with an NHAc group at C-2 exists as an α-anomer and is readily converted into α-H-phosphonate **268** under the action of chlorophosphite **129** (Scheme 20). Further improvement was achieved when the process was conducted in a microreactor, which provided a reduction in the reaction time to 30 min. and an increase in efficiency of the formation of α-H-phosphonate 268 to 76% [129].

In the synthesis of a mixture of the spacer-armed oligomer **250** with avDP12, the trimeric phosphoglycan **249** was extended using an enzymatic method. The mixture of trimer **249** and a nucleotide GlcNAc-UDP was passed through a HiTrap^®^ column with immobilized recombinant capsule polymerase CsxA [135], followed by fractionation of the resulting oligomers.

An advanced method of phosphonylation [134] of an anomeric mixture of hemiacetal **259** with chlorophosphite **129** was used by Indian researchers for the preparation of pseudo-tetrasaccharide **251** as an aminohexyl glycoside (Figure 12). Interaction of the hemiacetal mixture **259** with a phosphonylating agent (PhO)_2_POH in Py in the presence of Et_3_N, followed by treatment with H_3_PO_3_ for 4 days, afforded the desired α-H-phosphonate **261** with a 65% yield [134].

**Scheme 21:**
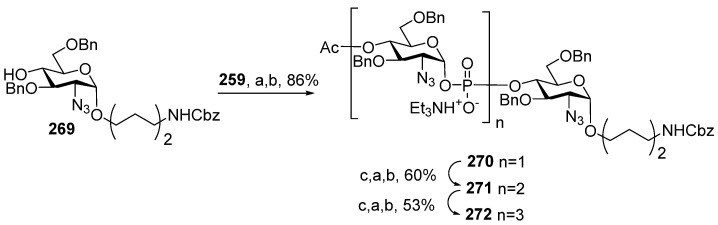
Key building blocks for the synthesis of pseudo-tetrasaccharide **251** (Figure 12) structurally related to *MenX* capsular phosphoglycans [134]. Reagents and conditions: (**a**) PivCl (2–3 eq. relative to 259), Py, 45 min; (**b**) 0.5 M I_2_ in Py:H_2_O 9.75:0.25; (**c**) MeONa/MeOH.

In the first step of the assembly of pseudo-tetrasaccharide **251**, the selectively protected aminohexyl glycoside **269** was condensed with H-phosphonate **259**, and after oxidation, the pseudo-tetrasaccharide **270** was formed. By sequential deacetylation, condensation with H-phosphonate **259**, and oxidation, the protected pseudo-tetrasaccharide **271** was obtained and further deblocked to yield the target pseudo-tetrasaccharide **271** (Scheme 21). In the course of the synthesis, the efficiency of the formation of the phosphodiester linkage decreased in each subsequent step (yields of 86% > 60% > 53%) [134], reflecting the tendency of the phosphodiester bond to be destroyed in the conditions of the H-phosphonate procedure.

**Figure 13:**
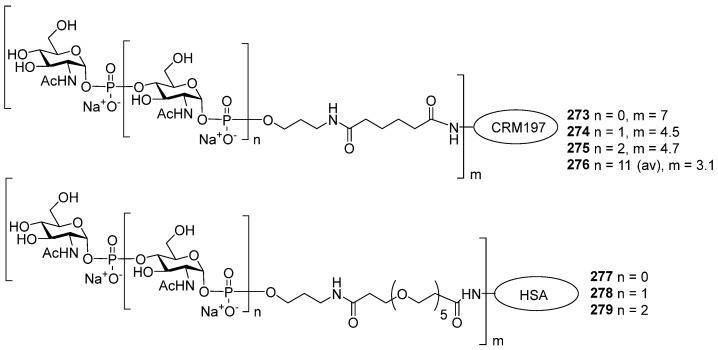
Neoglycoconjugates **273**–**275** prepared using synthetic *MenX*-related antigens **247**–**249** and CRM197 protein carrier and corresponding neoglycoconjugates **277**–**279** with HSA protein carrier [129].

The spacer-armed pseudo-saccharides **247**–**251** (Figure 12) were conjugated to protein carriers, and the immunogenic and antigenic properties of the resulting neoglycoconjugates were studied. Oligomers **247**–**249** were attached to CRM197 to yield conjugates **273**–**275** (Figure 13) and the corresponding conjugates **277**–**279** with HSA (Figure 13). Conjugates **273**–**275** were used for the immunization of mice at a dose of 0.3 μg of glycan per mouse. In addition, conjugate **275** was used at a dose of 1 μg per mouse. For positive controls, a group of mice was immunized with a conjugate of the partially fragmented *MenX* phosphoglycan avDP15 with CRM197 (*MenX*DP15-CRM197) at a dose of 0.3 μg and 1 μg of phosphoglycans, and bacterial MenX phosphoglycans were used as a coating antigen in ELISA experiments.

Conjugates **273** and **274** did not induce anti-*MenX* IgG antibodies. Immunization with conjugate **275** with trimeric antigens and *MenX*DP15-CRM197 efficiently elicited anti-*MenX* IgG antibodies, yet the intensity of the specific immune response for conjugate **275** was significantly weaker. For these conjugates, the immune response did not depend on the dose [133].

In the ELISA experiments, the immunogenic capacity of conjugates **273**–**275** was assessed using HSA conjugates **277**–**279** as coating antigens. It was found that the hyperimmune antisera of mice immunized with conjugates **273** (monomeric ligand) and **274** (dimeric ligand) contained negligible amounts of IgG antibodies to synthetic antigens. In contrast, immunization with conjugate **279** elicited anti-trimer IgG antibodies. An assessment of bactericidal activity in the rSBA tests showed that the pooled mouse sera obtained from animals immunized with conjugates **273** and **274** revealed that these conjugates did not elicit bactericidal antibodies. For conjugate **276**, rSBA titers were 16 times lower compared to *MenX*DP15-CRM197. Therefore, the length of the synthetic oligomers **247**–**250** (Figure 12), which are incorporated into conjugates **273**–**275**, was too small to effectively induce an anti-*MenX* immune response [133].

The mixture of the spacer-armed oligomer **250** with avDP12 was conjugated with the protein carrier CRM197. The resulting conjugate **276** was used for the immunization of mice, and the group of mice immunized with *MenX*DP15-CRM197 was used as a positive control. The antigenicity of conjugates **276** and *MenX*DP15-CRM197 was assessed in ELISA experiments with *MenX* capsular phosphoglycans as a coating antigen. It was found that conjugates **276** and *MenX*DP15-CRM197 demonstrate similar antigenic properties. However, SBA titers evidence the slightly greater bactericidal activity of antibodies elicited by *MenX*DP15-CRM197 [135].

## 7. Synthesis of Pseudo-Oligosaccharides Structurally Related to Phosphoglycans of *S. Pneumonia*

On a global scale, one in five infants under 1 year of age has had pneumonia, and ¾ of these pneumonia cases are caused by bacteria belonging to the *S. pneumoniae* species [136]. The prevention of community-acquired diseases associated with the most virulent S. pneumoniae serotypes is provided by a number of polyvalent conjugated pneumococcal vaccines, which comprise a number of pneumococcal capsular glycans conjugated to protein carriers.

Today, the pharmaceutical industry provides a whole range of anti-pneumococcal conjugated vaccines, including PCV7 (the *S. pneumoniae* serotypes 4, 6B, 9V, 14, 19F, 18C, and 23F), PCV10 (the *S. pneumoniae* serotypes 1, 4, 5, 6B, 7F, 9V, 14, 18C, 19F, and 23F) and (the *S. pneumoniae* serotypes 1, 5, 6A, 6B, 7F, 9V, 14, 19A, 19F, and 23F), PCV13 (the *S. pneumoniae* serotypes 1, 3, 4, 5, 6A, 6B, 7F, 9V, 14, 19A, 19F, 18C, and 23F), PCV15 (the *S. pneumoniae* serotypes 1, 3, 4, 5, 6A, 6B, 7F, 9V, 14, 18C, 19A, 19F, 22F, 23F, and 33F), and PCV20 (*S. pneumoniae* 1, 3, 4, 5, 6A, 6B, 7F, 8, 9V, 10A, 11A, 12F, 14, 15B, 18C, 19A, 19F, 22F, 23F, and 33F). Among the capsular glycan antigens used in these vaccines, five biopolymers, in particular *S. pneumoniae* 6A, *S. pneumoniae* 6B, *S. pneumoniae* 10A, *S. pneumoniae* 19A, and *S. pneumoniae* 19F, comprise phosphodiester bonds in the main chain. *S. pneumoniae* 6A and *S. pneumoniae* 6B phosphoglycans are built of pseudo-tetrasaccharide repeating units connected by phosphodiester linkage. The repeating unit of *S. pneumoniae* 10A phosphoglycans is a pseudo-heptaglycosyl phosphate, and the phosphoglycans of *S. pneumoniae* 19A and *S. pneumoniae* 19F are composed of trisaccharide phosphate and hexasaccharide phosphate repeating units [137].

Although the anti-pneumococcal vaccines for the prevention of *S. pneumoniae* 6A, *S. pneumoniae* 6B, *S. pneumoniae* 19A, and *S. pneumoniae* 19F have been available for more than 10 years, these serotypes are still frequently identified as the causative agents in pneumococcal pneumonia [136] and meningitis [138] and colonize the upper respiratory tract as opportunistic pathogens [139]. One of the factors hindering the broad coverage of people with anti-pneumococcal vaccination programs is high costs [9], which can be reduced by the replacement of bacterial antigens with their synthetic analogs [22].

The *S. pneumoniae* strains of serotype 6 are the common causative agents of invasive disease. The group is subdivided into serotypes 6A and 6B, which both have a capsule composed of phosphoglycans with Rha–ribitol–Gal–Glu subunits, which differ in the type of Rha–ribitol linkage. In serotype 6A, it is Rha1→3ribitol, and in serotype 6B, it is Rha1→4ribitol. It was reported that point mutation between *S. pneumoniae* 6A and *S. pneumoniae* 6B, as well as recombination, can compromise the linkage specificity and mediate serotype change [140]. As a result, it is reasonable to include both glycan antigens in combined vaccines. The surveillance of invasive pneumococcal disease in different countries evidences the immense importance of pneumococcal conjugated vaccines in the prevention of *S. pneumoniae* 6a- and *S. pneumoniae* 6b-associated invasive diseases [139,141,142]. In contrast, in regions with low immunization coverage [143], this pathogen makes a substantial contribution to the burden of invasive pneumonia.

**Figure 14:**
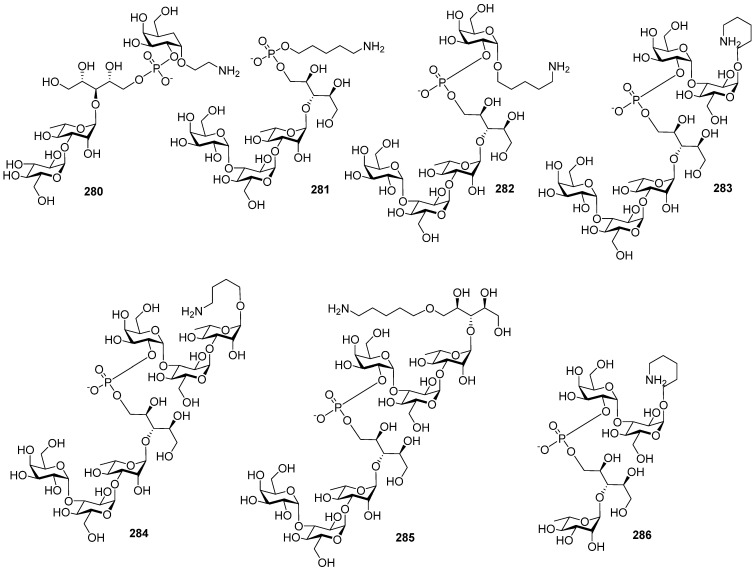
Synthetic pseudo-tetrasaccharide **280** [144] and the series of pseudo-oligosaccharides **281**–**286** [145] structurally related to *S. pneumoniae* 6A capsular phosphoglycans.

**Scheme 22:**
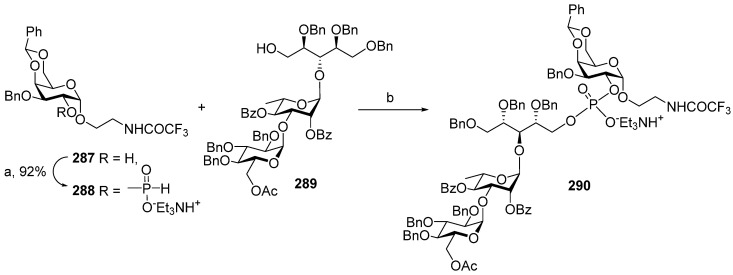
Key building blocks for the synthesis of pseudo-oligosaccharide 280 structurally related to *S. pneumoniae* 6A capsular phosphoglycans [144]. Reagents and conditions: (**a**) PivCl, Py, H_3_PO_3_, 92%; (**b**) PivCl (4.8 eq), Py, 3 h, followed by 0.5 M I_2_ in Py:H_2_O 2:1, yield over 2 steps at 73%.

Recently, syntheses of the spacer-armed pseudo-oligosaccharides **280**–**286** [144,145] structurally related to *S. pneumoniae* 6A phosphoglycans (Figure 14) have been reported. For the construction of a phosphodiester bond in compound **280**, Nifantiev et al. [144] used the H-phosphonate procedure. Glycoside **287** (Scheme 22) was converted into H-phosphonate **288** by treatment with H_3_PO_3_ in Py and the presence of PivCl. The condensation of H-phosphonate **288** with the primary alcohol **289**, followed by oxidation, afforded phosphodiester **290**, which, after removal of the protective groups, was converted into aminoethyl glycoside **280**.

**Scheme 23:**
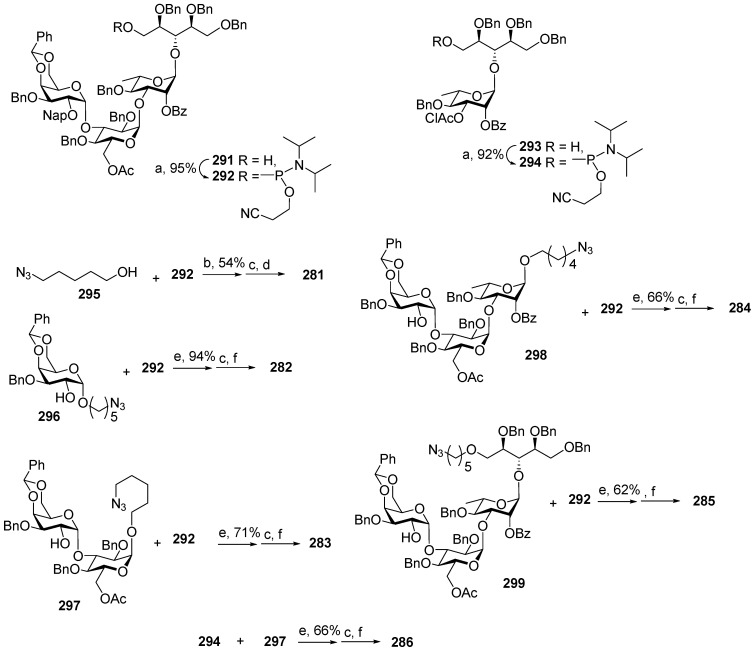
Key building blocks for the synthesis of pseudo-oligosaccharides **281**–**286** structurally related to *S. pneumoniae* 6A capsular phosphoglycans [145]. Reagents and conditions: (**a**) diamidite 70, diisopropylammonium tetrazolide; (**b**) 1H-tetrazole, CH_2_Cl_2_, r.t., 1 h, followed by mCPBA, −20 °C, 30 min; (**c**) Bu_4_NOH, CH_2_Cl_2_, H_2_O, r.t., 4 h; (**d**) Li/NH_3_, THF, −78 °C, 1 h, followed by MeOH, 16 h; (**e**) 5-ethylthio-1H-tetrazole, 3 Å MS, CH_3_CN, r.t., 1 h, followed by H_2_O, I_2_/THF, r.t., 2 h; (**f**) 0.2 M NaOMe/MeOH, followed by Pd(OH)_2_/C, H_2_, MeOH, H_2_O, AcOH.

Pseudo-oligosaccharides **281**–**286** were assembled by Taiwan scientists [145] using a phosphoramidite procedure for the establishment of phosphodiester linkage (Scheme 23). The selectively protected pseudo-tetrasaccharide **291** with a primary hydroxyl group in the ribitol residue was transformed into phosphoramidite **292** by the action of diamidite **70** and diisopropylammonium tetrazolide. In similar conditions, pseudo-disaccharide **293** was converted into phosphoramidite **294**.

The interaction of the key phosphoramidite **292** with 5-azidopentanol (compound **295**) in the presence of 1H-tetrazole, followed by oxidation with mCPBA, the removal of the protective groups, and the reduction of the azido group, resulted in pseudo-tetrasaccharide **281** (Scheme 23). The condensation of phosphoramidite **292** with alcohols **296**–**299** was carried out in the presence of 5-ethylthio-1H-tetrazole, followed by oxidation of a phosphite group to a phosphotriester with I_2_ in THF. The efficiency of the formation of the phosphotriester linkage decreased in the sequence of monosaccharide **296** (94%), disaccharide **297** (71%), trisaccharide **298** (66%), and pseudo-tetrasaccharide **299** (62%), along with an increase in the steric hindrance of the hydroxyl group involved in condensation. The reduction and deblocking of the obtained phosphotriesters afforded the amino-spacered derivatives **282**–**285**. The condensation of phosphoramidite **294** with disaccharide **297**, followed by oxidation, the reduction of the azido group, and deblocking, formed pseudo-tetrasaccharide **286** [145].

**Figure 15:**
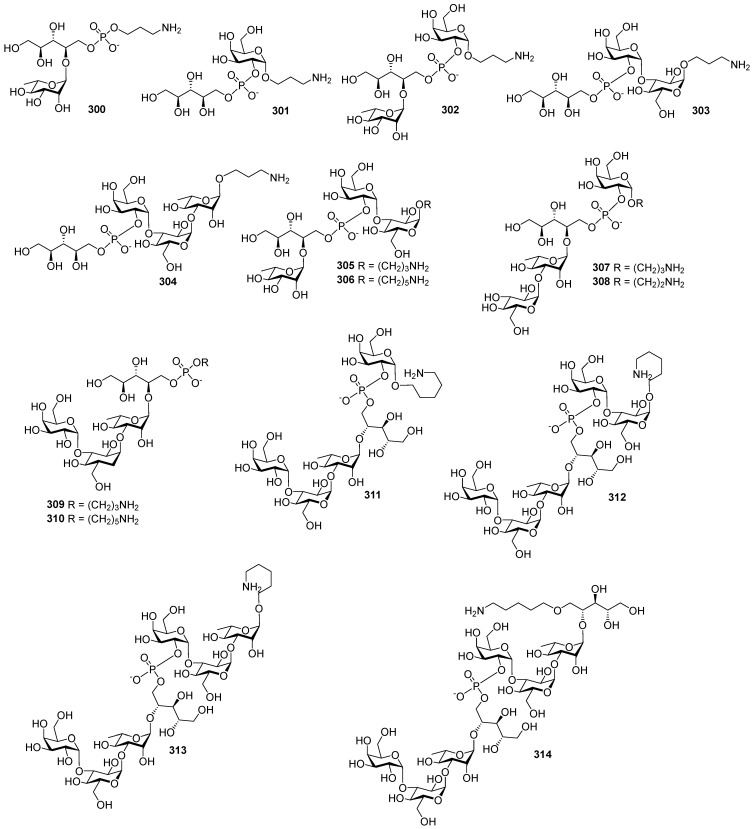
Synthetic oligosaccharides **300**–**303** [146], **304**, **305**, **307**, **309**, and **309** [147], **308** [148], and **306** and **310**–**314** [145] related to *S. pneumoniae* 6B capsular phosphoglycans.

Due to interest in the development of vaccine preparations based on the synthetic phosphooligosaccharides related to *S. pneumoniae* 6B, extensive libraries of the spacer-armed pseudo-oligosaccharides **300**–**314** [145,146,147,148] (Figure 15) related to *S. pneumoniae* 6B CPS fragments have been created. Despite their structural diversity, these compounds comprise only one phosphodiester linkage that is connected with the synthetic complexity of the target molecules.

**Scheme 24:**
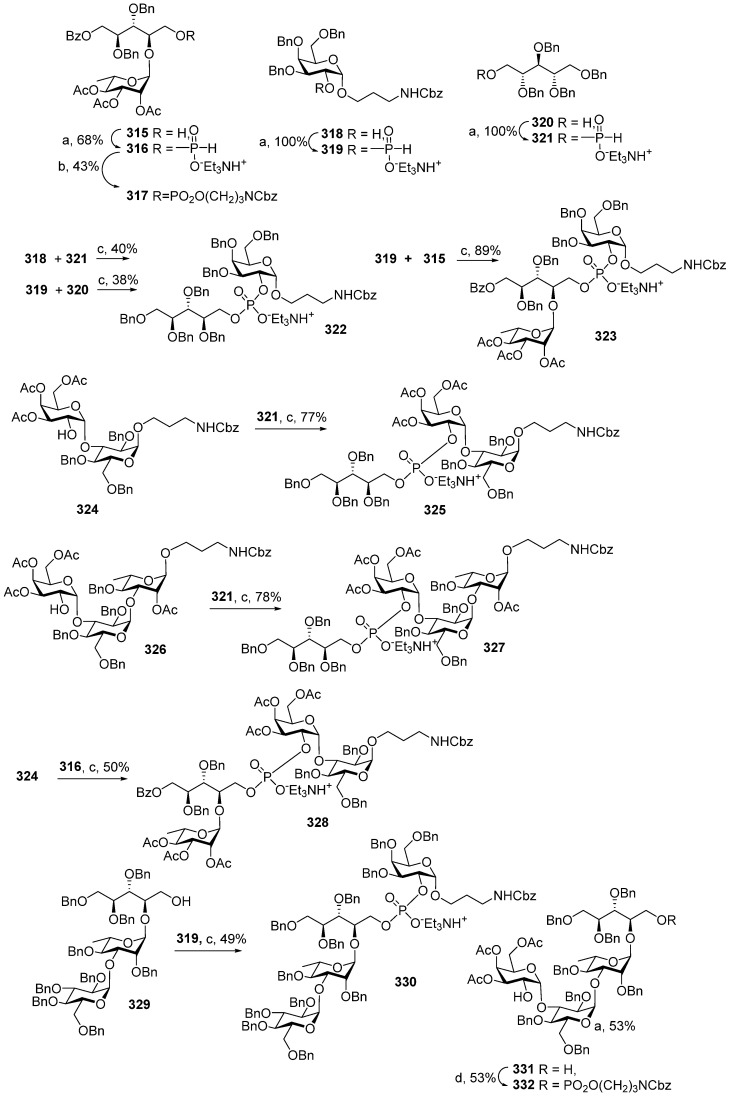
Key building blocks for the synthesis of pseudo-oligosaccharides **300**–**304**, **305**, and **307** structurally related to *S. pneumoniae* 6B capsular phosphoglycans [146,147]. Reagents and conditions: (**a**) **129**, Py, 24 h; (**b**) HOCH_2_CH_2_CH_2_CH_2_NHCbz, PivCl, Py, followed by 0.5 M I_2_ in Py:H_2_O 95:5; 20 min; (**c**) PivCl, Py, followed by 0.5 M I_2_ in Py:H_2_O 95:5, 20 min; (**d**) **214**, PivCl, Py, followed by 0.5 M I_2_ in Py:H_2_O 95:5, 20 min.

The assembly of the spacer-armed pseudo-disaccharides **300** and **301** and pseudo-trisaccharides **302** and **303** was carried out by Vliegenthart [146] using the H-phosphonate method. For the preparation of pseudo-disaccharide **300**, the starting pseudo-disaccharide **315** was converted to the key H-phosphonate **316** by the action of chlorophosphite **129** (Scheme 24). H-phosphonate **316** was condensed with N-protected aminopropanol in the presence of PivCl in Py, the product was oxidized, and a phosphodiester **317** was obtained with a 43% yield. The deprotection of phosphodiester **317** yielded the target spacer-armed pseudo-disaccharide **300**.

**Scheme 25:**
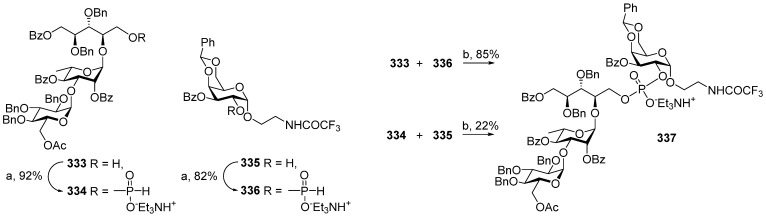
Key building blocks for the synthesis of pseudo-oligosaccharide **308** structurally related to *S. pneumoniae* 6B phosphoglycans [148]. Reagents and conditions: (**a**) **129**, Py, 1 h; (**b**) PivCl, Py, 2 h, followed by I_2_ in Py:H_2_O 2:1, 4 h.

Under similar conditions, alcohol **318** (Scheme 25) was transformed into H-phosphonate **319**, and the phosphonylation of alcohol **320** produced H-phosphonate **321** with an excellent yield. Phosphodiester **322** was prepared via two alternative routes. The condensation of the secondary hydroxyl group in galactoside **318** with H-phosphonate **321**, followed by oxidation, formed **322** with a 40% yield, and the interaction of a primary hydroxyl group in the selectively protected ribitol **320** with H-phosphonate **319** and oxidation of the intermediate phosphonate afforded pseudo-disaccharide **322** with a 38% yield. By the removal of the benzyl and Cbz groups, compound **322** was converted into the target pseudo-disaccharide **301** (Figure 15) [146].

Unlike the synthesis of pseudo-disaccharide **322**, the phosphitylation of alcohol **315** with H-phosphonate **319** was more efficient and readily formed pseudo-trisaccharide **323** (yield: 89%) [147], which was deprotected to yield the target spacer-armed pseudo-trisaccharide **302** (Scheme 24) [146]. The substantial difference in the yields for pseudo-disaccharide **322** (from **319** and **320**) and pseudo-trisaccharide **323** may be related to the easier accessibility of a less sterically hindered phosphodiester bond in pseudo-disaccharide **322** for the I_2_-induced rapid rupture of the bridging P-O bonds. The total deprotection of pseudo-trisaccharide **323** resulted in the formation of the target spacer-armed pseudo-trisaccharide **302** (Figure 15) [147].

The condensation of alcohol **324** with H-phosphonate **321** (Scheme 24), followed by oxidation, afforded pseudo-tetrasaccharide **325** with a 77% yield [147]. Similar to the phosphitylation of alcohols **315** and **320** with H-phosphonate **319**, the improved efficiency of preparation of more sterically hindered pseudo-tetrasaccharide **325** compared to **322** (from **318** and **321**, 40%) [146] can be attributed to the easier destruction with I_2_ during the oxidation step. By deprotection, pseudo-trisaccharide **325** was converted into the target spacer-armed pseudo-trisaccharide **303** (Figure 15) [147].

The interaction of H-phosphonate **321** with the trisaccharide alcohol **326** (Scheme 24), followed by oxidation, formed pseudo-tetrasaccharide **327** (78%) with a phosphodiester bond between a bulky trisaccharide and a flexible ribitol residue. On the contrary, the efficiency of the formation of pseudo-tetrasaccharide **328** (from disaccharide **324**) and pseudo-tetrasaccharide **330** (from pseudo-tetrasaccharide **329**), which comprise bulky protected glycoside residues on both sides of a phosphate moiety, was much lower, and the yields did not exceed 50%. The total deprotection of pseudo-tetrasaccharides **328** and **329** resulted in the target spacer-armed compounds **304** and **305**, respectively [147].

The condensation of pseudo-tetrasaccharide **331** with H-phosphonate **214**, followed by oxidation, provided the introduction of a pre-spacer group, which is connected to the pseudo-tetrasaccharide via a phosphodiester bridge. The moderate yield of compound **332** (53%) can be attributed to the conformational flexibility of the pre-spacer group and the easier availability of the phosphate group for the action of I_2_. Compounds **331** and **332** were deblocked to yield the target spacer-armed pseudo-tetrasaccharides **307** and **309**, respectively (Scheme 24) [147].

For the assembly of pseudo-tetrasaccharide **308**, Nifantiev et al. [148] investigated two alternative pathways according to the schemes [3 + 1], which differed in the position of the H-phosphonate group on one or the other condensing part (Scheme 25). For this purpose, alcohol **333** was converted into H-phosphonate **334** by the action of chlorophosphite **129**, and galactoside **335** was converted into H-phosphonate **336** under similar conditions. The condensation of H-phosphonate **336** with the primary hydroxyl group of pseudo-tetrasaccharide acceptor **333**, followed by oxidation, produced phosphodiester **337** in a high yield (85%), whereas the interaction of H-phosphonate **334** with the secondary hydroxyl group in galactoside **335** proceeded with low efficiency (the yield of pseudo-tetrasaccharide **337** was 22%).

**Scheme 26:**
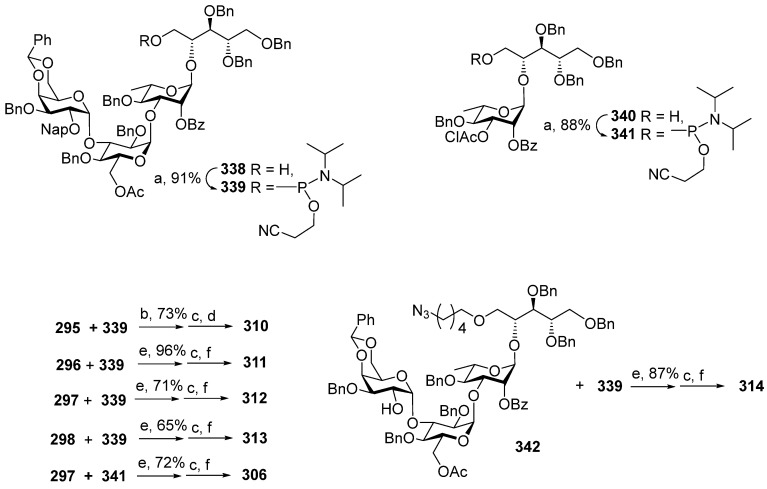
Key building blocks for the synthesis of pseudo-oligosaccharides **281**–**286** structurally related to *S. pneumoniae* 6B phosphoglycans [145]. Reagents and conditions: (**a**) diamidite **70**, diisopropylammonium tetrazolide; (**b**) 1H-tetrazole, CH_2_Cl_2_, r.t., 1.5 h, followed by mCPBA, −20 °C, 30 min; (**c**) Bu_4_NOH, CH_2_Cl_2_, H_2_O, r.t.; (**d**) Li/NH_3_, THF, −78 °C, 1 h, followed by MeOH, 16 h; (**e**) 5-ethylthio-1H-tetrazole, molecular sieves 3Å, CH_3_CN, r.t., 1 h, followed by H_2_O, I_2_/THF, r.t., 2 h; (**f**) 0.2 M NaOMe/MeOH, followed by Pd(OH)_2_/C, H_2_, MeOH, H_2_O, AcOH.

For the preparation of pseudo-oligosaccharides **306** and **310**–**314 [145]**, Taiwan researchers used the universal pseudo-tetrasaccharide block **338** (Scheme 26), which was readily converted into the corresponding phosphoramidite **339** by the action of diamidite **70** in the presence of diisopropylammonium tetrazolide. In similar conditions, pseudo-disaccharide **340** was converted into phosphoramidite **341**. The condensation of alcohols **295**–**298** and **342** with compound **339** added a repeating unit to the chains. Thus, the phosphitylation of the primary hydroxyl group in alcohol **295** with phosphoramidite **339** in the presence of 1H-tetrazole, followed by the oxidation of phosphite to phosphate and the removal of the 2-naphthylmethyl protective group, afforded alcohol **342**, and the total deprotection and reduction of the azido group resulted in the target spacer-armed pseudo-tetrasaccharide **310**, with the spacer group connected to the pseudo-tetrasaccharide by a phosphodiester bridge.

In a similar way, phosphoramidite **339** readily phosphitylated secondary hydroxyl groups in monosaccharide **296**, disaccharide **297**, and trisaccharide **298** (Scheme 26). The resulting phosphites were oxidized and deprotected to obtain target compounds **311**–**313**. The condensation of two pseudo-tetrasaccharides **342** and **339**, followed by oxidation, afforded a pseudo-octasaccharide (87%), with two bulky fragments connected by a phosphodiester bridge. After the deprotection and reduction of the azido group, the spacer-armed pseudo-octasaccharide **314** was obtained. Disaccharide **297** was phosphitylated with phosphoramidite **341**. The oxidation of the intermediate phosphite, removal of the protective groups, and reduction of the azido group afforded pseudo-tetrasaccharide **306**. It can be concluded that the use of phosphoramidites as phosphitylating agents in the preparation of compounds **306** and **310**–**314** was efficient regardless of the steric demands of the secondary hydroxyl group in the acceptor molecule. This result shows that the phosphoramidite method is preferred for the synthesis of larger structures.

**Figure 16:**
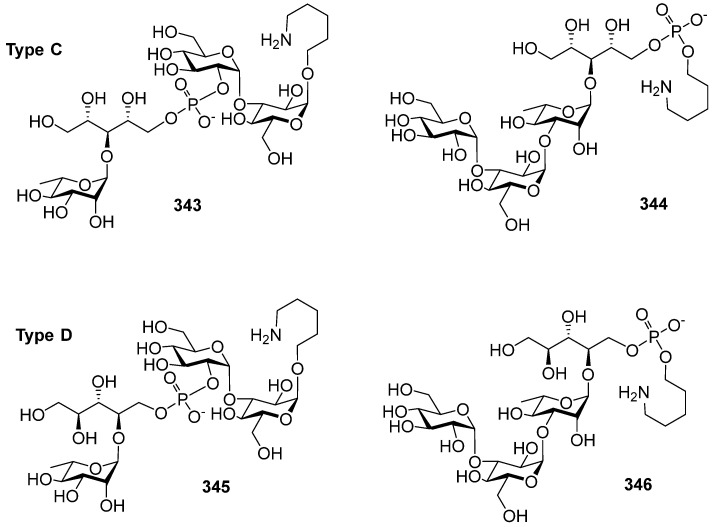
Synthetic phosphooligosaccharides related to *S. pneumoniae* 6C (compounds **343** and **344**) and *S. pneumoniae* 6D (compounds **345** and **346**) capsular phosphoglycans [151].

**Scheme 27:**
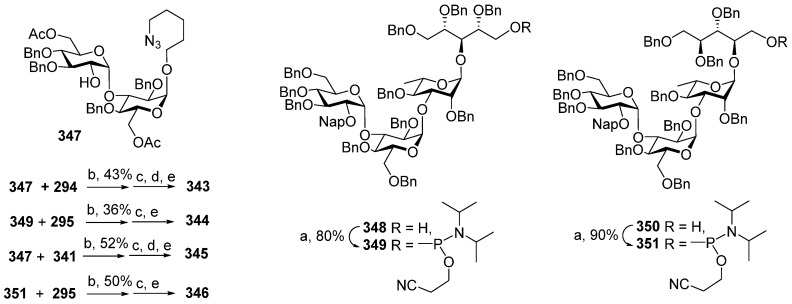
Key building blocks for the synthesis of pseudo-oligosaccharides **343** and **344**, structurally related to *S. pneumoniae* 6C phosphoglycans, and pseudo-oligosaccharides **345** and **346**, structurally related to *S. pneumoniae* 6D phosphoglycans [151]. Reagents and conditions: (**a**) diamidite **70**, diisopropylammonium tetrazolide, 1 h; (**b**) 5-ethylthio-1H-tetrazole, molecular sieves 3Å, CH_3_CN, r.t., 1 h, followed by H_2_O, I_2_/THF, r.t., 2 h; (**c**) Bu_4_NOH, CH_2_Cl_2_, H_2_O, r.t.; (**d**) followed by 0,3 M NaOMe, MeOH, CH_2_Cl_2_, 2 h; (**e**) Pd(OH)_2_/C, H_2_, MeOH, H_2_O, AcOH, 36 h.

The phosphoglycan structure of *S. pneumoniae* 6C differs from *S. pneumoniae* 6A in the orientation of a single hydroxyl group in one of the monosaccharide blocks (Glc in *S. pneumoniae* 6C vs. Gal in *S. pneumoniae* 6A), and a similar structural difference is observed between *S. pneumoniae* 6D and *S. pneumoniae* 6B (Figure 16). It is generally accepted that a close structural resemblance gives rise to similar antigenic properties, and one could expect cross-reactivity between all serotypes in group *S. pneumoniae* 6. However, surveillance data and clinical efficacy studies for subtypes of *S. pneumoniae* 6 obtained in connection with the use of pneumococcal vaccines are controversial. Generally, cross-protection was observed from the *S. pneumoniae* 6B conjugate in PCV7 and PCV10 against *S. pneumoniae* 6A but not against *S. pneumoniae* 6C and *S. pneumoniae* 6D. However, it was shown that PCV13, which comprises the *S. pneumoniae* 6A antigen, was effective against serotypes 6C and 6D invasive pneumococcal disease and microbial carriage [149,150]. The challenges associated with the assessment of the cross-reactivity and cross-protection of *S. pneumoniae* group 6 could be met via the identification of a glycotope for each *S. pneumoniae* 6 subtype. To this end, the series of oligosaccharides **343** and **344**, structurally related to *S. pneumoniae* 6C, and **345** and **346**, structurally related to *S. pneumoniae* 6D, were synthesized (Figure 16) [151]. The preparation of pseudo-tetrasaccharides **343** and **344**, which comprise phosphodiester linkages, was performed using phosphoramidite chemistry. To obtain pseudo-oligosaccharide **343** with an interglycosidic phosphodiester bridge, disaccharide **347** was reacted with phosphoramidite **294** in acetonitrile in the presence of 5-ethylthio-1H-tetrazole (Scheme 27). The reaction mixture was oxidized under the action of iodine in THF with the addition of water, the protective groups were removed, and the N_3_ group was reduced to NH_2_. The phosphitylation of the primary OH-group in pseudo-tetrasaccharide **348** with diamidite **70** resulted in the efficient formation of phosphoramidite **349**. The condensation of phosphoramidite **349** with alcohol **295**, followed by the reduction of the N_3_ group and total deprotection, afforded pseudo-tetrasaccharide **344**, in which a phosphodiester linkage connects the ribitol part and a spacer [151].

In a similar way, pseudo-tetrasaccharide **345** with an interglycosidic phosphodiester bridge was obtained by the condensation of disaccharide **247** and phosphoramidite **341**, oxidation, reduction of the N_3_ group, and deprotection. For the preparation of phosphodiester **346**, pseudo-tetrasaccharide **350** was transformed into phosphoramidite **351**, which was then condensed with alcohol **295**. After oxidation, the condensation product was reduced and deprotected to yield pseudo-tetrasaccharide **346**, with a phosphodiester bridge between ribitol and a spacer. Notably, in the preparation of compounds **343**–**346**, the corresponding protected phosphodiesters were obtained in moderate yields regardless of the position of the phosphodiester bridge in the molecule. It can be concluded that the used method is not sensitive to steric factors and can be applied for the condensation of bulky counterparts.

The cross-reactivity of *S. pneumoniae* 6A and *S. pneumoniae* 6B was evaluated in an immunological study of pseudo-tetrasaccharides **280** [144] related to *S. pneumoniae* 6A (Figure 14) and **308** [148] (Figure 15) related to *S. pneumoniae* 6B. Conjugates of these compounds with BSA (**280**-BSA and **308**-BSA, respectively) were used for the immunization of mice at a dose of 20 μg/mouse on days 0 and 14. The hyperimmune serum obtained after immunization with **280**-BSA and **308**-BSA was analyzed in ELISA experiments using the corresponding N-biotinylated conjugates **352** and **353** on streptavidin-coated plates. It was shown that the antibodies induced by the *S. pneumoniae* 6A-related conjugate **280**-BSA recognized not only the *S. pneumoniae* 6A-related N-biotinylated conjugate **352** but also the *S. pneumoniae* 6B-related N-biotinylated conjugate **353**. Inversely, the specificity of the serum obtained from mice immunized with the *S. pneumoniae* 6B-related conjugate **308**-BSA to the *S. pneumoniae* 6A-related conjugate **352** was detected. These results evidence the cross-reactivity of pseudo-tetrasaccharides **280** and **308** and indicate the presence of a common epitope in these antigens.

The immunogenic properties of synthetic phosphooligosaccharides **285** and **286** related to *S. pneumoniae* 6A phosphoglycans (Figure 14) and phosphooligosaccharides **306** and **314** related to *S. pneumoniae* 6B phosphoglycans were studied. The conjugates of these compounds with CRM197 were obtained and used for the immunization of mice at a dose of 2.2 μg of glycan per mouse, with three shots in two-week intervals. The glycan-specific IgG in the serum was analyzed using a microarray with a number of synthetic glycan antigens [145]. Mice in the negative control group were immunized with CRM197. It was found that conjugates **286**-CRM197 and **306**-CRM197, which contain pseudo-tetrasaccharides, effectively induced IgG antibodies against the majority of other synthetic antigens. On the contrary, conjugates **285**-CRM197 and **314**-CRM197 showed low immunogenicity [145].

**Figure 17:**
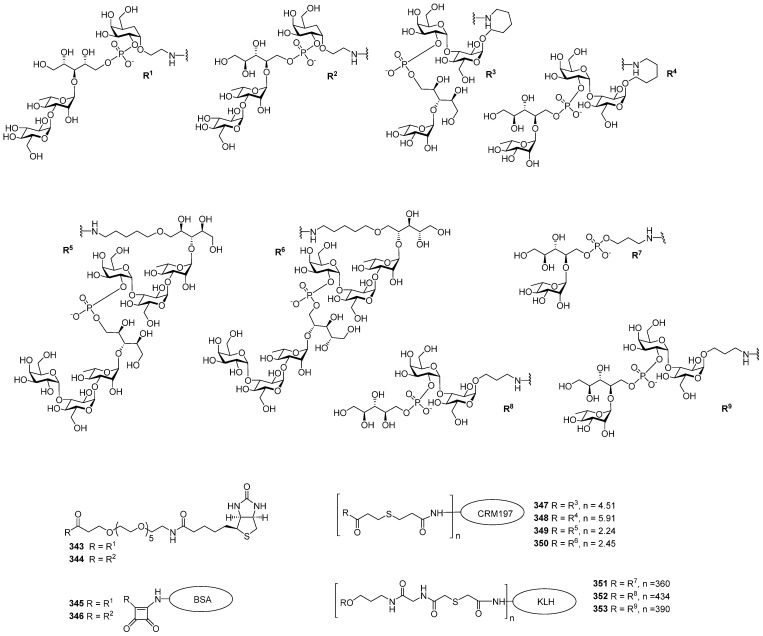
Neoglycoconjugates **343**–**353** obtained on the basis of synthetic pseudo-oligosaccharides structurally related to *S. pneumoniae* 6A and *S. pneumoniae* 6B capsular phosphoglycans.

The antigenic properties of *S. pneumoniae* 6B-related pseudo-disaccharide **301**, pseudo-trisaccharide **303**, and pseudo-tetrasaccharide **305** were investigated using their conjugates **301**-KLH–**305**-KLH with the highly immunogenic protein KLH (Figure 17) [152]. The neoglycoconjugates **301**-KLH–**305**-KLH and a conjugate obtained by the condensation of bacterial *S. pneumoniae* 6B phosphoglycans and KLH (*S. pneumoniae* 6B-KLH) were used for the immunization of mice at a dose of 2.5 μg and rabbits at a dose of 10 μg. The hyperimmune sera were analyzed in ELISA experiments with bacterial *S. pneumoniae* 6B and *S. pneumoniae* 6A phosphoglycans. Human serum samples were obtained by pooling infant antisera obtained by vaccination with PCV7-CRM197 at a dose of 4 mg of 6B PS.

It was shown that in mice conjugates, **301**-KLH with pseudo-disaccharide antigens and **303**-KLH with pseudo-trisaccharide antigens were poorly immunogenic. On the contrary, the conjugate **305**-KLH was more immunogenic than *S. pneumoniae* 6B-KLH. ELISA analysis of the rabbit hyperimmune antisera using bacterial *S. pneumoniae* 6A phosphoglycans as a coating antigen showed that conjugates **303**-KLH and **305**-KLH were able to induce anti-*S. pneumoniae* 6A antibodies. Antibody specificity was confirmed in ELISA inhibition and phagocytosis experiments. The binding of human PCV7-CRM197 antisera to the conjugates **301**-KLH–**305**-KLH was inhibited by bacterial *S. pneumoniae* 6A and *S. pneumoniae* 6B phosphooligosaccharides.

**Scheme 28:**
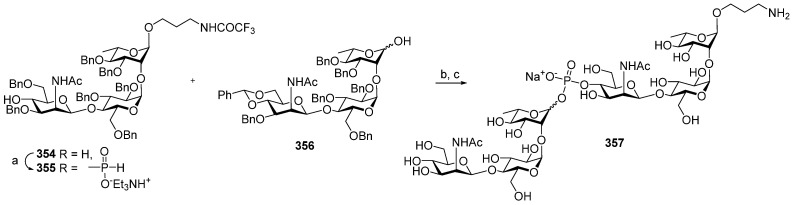
Key building blocks for the synthesis of pseudo-oligosaccharide **357** structurally related to *S. pneumoniae* 19F capsular glycan [154]. Reagents and conditions: (**a**) **129**, acetonitrile, Py, 1 h.; (**b**) PivCl, Py, then I_2_ B Py:H_2_O 19:1, yield 61% over 3 steps; (**c**) H_2_, Pd(OH)_2_/C, MeOH–H_2_O.

*S. pneumoniae* 19F is one of the most virulent pneumococcal serotypes. Similarly to *S. pneumoniae* 6A and *S. pneumoniae* 6B, the diseases caused by *S. pneumoniae* 19F infection are associated with increased mortality and morbidity [153] despite the fact that the *S. pneumoniae* 19F capsular glycan is a component of many pneumococcal conjugated commercial vaccines. In light of this, it could be useful to develop an anti-*S. pneumoniae* 19F conjugate vaccine with a synthetic glycan antigen structurally related to the capsular glycan of *S. pneumoniae* 19F. N-trifuoroacetamidopropyl glycoside **357**, which comprises a pseudo-hexasaccharide fragment of the *S. pneumoniae* 19F capsular polysaccharide, was obtained as a mixture of isomers. In the first step, the selectively protected trisaccharide **354** with a free hydroxyl group at C-4 (ManNAc) was converted into H-phosphonate **355** under the action of chlorophosphite **129** in Py [154] (Scheme 28). The condensation of H-phosphonate **355** with hemiacetal **356**, followed by oxidation and deprotection, resulted in the formation of a diastereomeric mixture of pseudo-hexasaccharides, which contained the spacer-armed phosphooligosaccharide **357**.

The structure of the repeating unit of *S. pneumoniae* 19A is close to that of 19F, with the only difference being the type of linkage between Glc and Rha monosaccharide residues, which is α-D-Glc-(1→3)-Rha in *S. pneumoniae* 19A phosphoglycans and α-D-Glc-(1→2)-Rha in *S. pneumoniae* 19F phosphoglycans. Whereas *S. pneumoniae* 19F phosphoglycans are a component of all commercial PCV compositions, *S. pneumoniae* 19A phosphoglycans were not included in PCV7 and PCV10. After the introduction of PCV7, serotype replacement resulted in an increase in the number of invasive pneumococcal diseases caused by S. pneumoniae 19A [155,156]. This observation shows the absence of cross-protection between serotypes 19A and 19F. Nevertheless, the synthetic complexity of the repeating units of *S. pneumoniae* 19A and *S. pneumoniae* 19F phosphoglycans, which share a common structure, stimulated attempts to develop a universal synthetic glycoantigen for the induction of protective antibodies to both serotypes.

**Figure 18:**
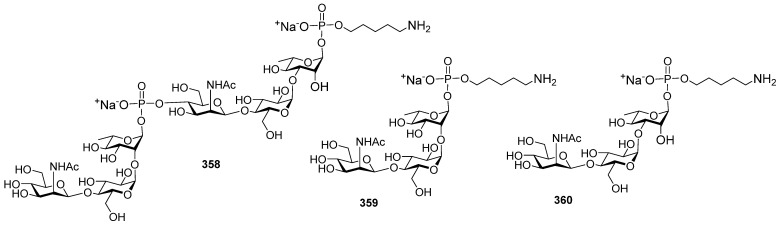
Synthetic phosphohexasaccharide hybrid antigen **358**, which comprises repeating units of *S. pneumoniae* 19F and *S. pneumoniae* 19A and phosphotrisaccharides related to *S. pneumoniae* 19F (compound **359**) and *S. pneumoniae* 19A capsular glycan (compound **360**) [157].

With a view to investigating the ability of a neoglycoconjugate with a chimeric synthetic antigen to induce a protective immune response, the Seeberger group prepared [157] phosphohexasaccharide **358**, which is a combination of *S. pneumoniae* 19A and S. pneumoniae 19F phosphoglycan repeating units in one molecule and the spacer-armed phosphotrisaccharides **359** and **360** related to repeating units of *S. pneumoniae* 19F and *S. pneumoniae* 19A, respectively (Figure 18).

**Scheme 29:**
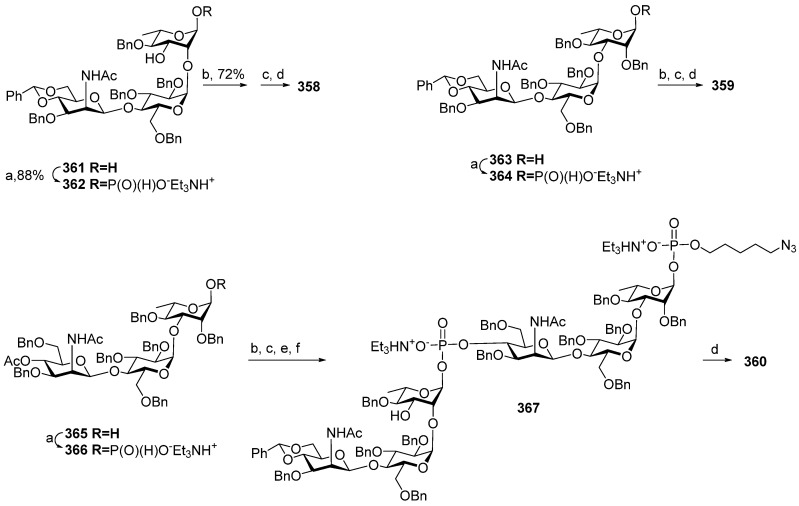
Key building blocks for the synthesis of pseudo-oligosaccharides **358**–**360** [157]. Reagents and conditions: (**a**) chlorophosphite **129**, H_3_PO_3_, Py, r.t, 2 h then 50 °C, 36 h; (**b**) alcohol **295**, PivCl, pyridine, r.t., 1.5 h; (**c**) I_2_, Py:H_2_O (18:2), −40 °C, 30 min, then Et_3_NHBr; (**d**) H_2_, Pd/C, EtOAc:MeOH:H_2_O, 3 days, then Dowex 50W X4, Na^+^; (**e**) 0.5 M NaOMe, MeOH, r.t., 3 h; (**f**) α-H-phosphonate **362**, PivCl, Py, r.t., 2 h.

Hemiacetal **361** was readily transformed into the corresponding α-H-phosphonate **362** under the action of chlorophosphite **129** and H_3_PO_3_ in Py (Scheme 29). The authors suggest that α-stereoselectivity was attained due to thermodynamic reaction control, which favored α-H-phosphonate **362** in the conditions of S_N_2 displacement with H_3_PO_3_ at the anomeric center. In a similar manner, hemiacetal **363** was stereoselectively converted into α-H-phosphonate **364** as a precursor of the *S. pneumoniae* 19A repeating unit. α-H-Phosphonates **362** and **364** were coupled with alcohol **295**, oxidized, reduced, and deprotected to yield the spacer-armed glycoantigens **359** and **360**, which correspond to repeating units of *S. pneumoniae* 19F and *S. pneumoniae* 19A phosphoglycans [157].

For the preparation of the hybrid antigen 358, another precursor of the *S. pneumoniae* 19A repeating unit, hemiacetal **365** with an acetyl protecting group at O-4 (MaNAc) was phosphonylated to obtain α-H-phosphonate **366**, which was then condensed with alcohol **295** and oxidized. The following 4-O-deacetylation and phosphitylation with α-H-phosphonate **362** and oxidation yielded the protected phosphodiester **367**. The deblocking and reduction of the azido group in phosphodiester **367** afforded the conjugation-ready chimeric phosphodiester 358 [157]. Phosphooligosaccharides **358**–**361** were conjugated to CRM197 via a succinimidyl adipate linker, and the resulting conjugates **358**-CRM197 (average glycan loading: 5), **359**-CRM197 (average glycan loading: 5), and **360**-CRM197 (average glycan loading: 7) were adsorbed on alum and used for the vaccination of rabbits on day 0 and boosted on days 14, 28, and 133. IgG antibodies were raised in response to immunization with the semisynthetic neoglycoconjugates **358**-CRM197, **359**-CRM197, **360**-CRM197, and Prevnar 13^®^ (PCV13).

Oligosaccharide-specific antibody titers were analyzed by glycan microarray with specific immobilized oligosaccharides. Chimeric **358**-CRM197 generated antibodies that recognized trisaccharide antigens **359** and **360** in quantities exceeding those generated by Prevnar 13^®^. At the same time, ELISA experiments showed that antibodies produced by vaccination with Prevnar 13^®^ more efficiently recognized *S. pneumoniae* 19A and *S. pneumoniae* 19F phosphoglycans compared to vaccine preparations with synthetic antigens. This observation reveals that upon immunization with Prevnar 13^®^, the antibodies are elicited to larger glycotopes. Immunization with chimeric **358**-CRM197 produced antibodies that efficiently recognized phosphotrisaccharide antigens **359** and **360** and *S. pneumoniae* 19A and *S. pneumoniae* 19F phosphoglycans, whereas antibodies elicited by **359**-CRM197 and **360**-CRM197 did not recognize *S. pneumoniae* 19A phosphoglycans, and only moderate interaction of **359**-CRM197-induced antibodies and S. pneumoniae 19F phosphoglycans was detected. Bactericidal properties of the obtained hyperimmune sera were studied in the opsonophagocytic killing assay. Chimeric **358**-CRM197-induced opsonic antibodies were able to kill both *S. pneumoniae* 19A and *S. pneumoniae* 19F. The opsonic activity of **359**-CRM197-induced antibodies was very weak, and antibodies induced by the ST19A conjugate were not bactericidal [157].

**Figure 19:**
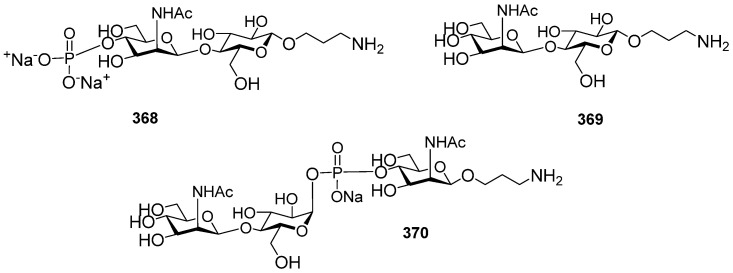
Synthetic glycans related to phosphodisaccharide P(1→4)ManNAc-β-(1→4)-Glc, which is a common structural element of *S. pneumoniae* 19A and *S. pneumoniae* 19F phosphoglycans.

As previously mentioned, clinically significant cross-protection between *S. pneumoniae* 19A and *S. pneumoniae* 19F serotypes has not been reported. One of the possible reasons for weak antibody cross-protection is the conformational difference between these two phosphoglycans [158]. In shorter oligosaccharides, the chain exists as another ensemble of conformers, which may be able to induce antibodies to phosphoglycans of both serotypes. The capsular phosphoglycans *S. pneumoniae* 19A and *S. pneumoniae* 19F share a common disaccharide structural element: P(1→4)ManNAc-β-(1→4)-Glc. With a view to studying the universal glycotope for these serotypes, a series of short glycans **368**–**370** (Figure 19) were prepared, which comprise the basic structural elements of the common disaccharide.

**Scheme 30:**
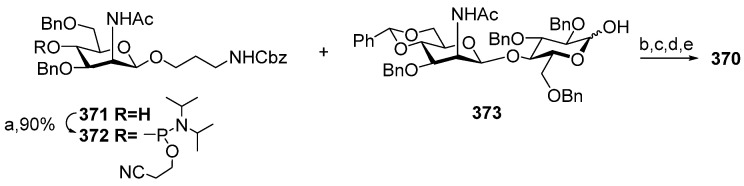
Key building blocks for the synthesis of pseudo-trisaccharide **370** [159]. Reagents and conditions: (**a**) **24**, DIPEA, CH_2_Cl_2_, 90%; (**b**) DCI, CH_2_Cl_2_, 95%; (**c**) tBuOOH, CH_3_CN, 0 °C to r.t., 73% (55% α); (**d**) Et_3_N, DCM, 4 days; (**e**) Pd/C, H_2_, MeOH/H_2_O, 90%.

The phosphoramidite approach was applied for the construction of the phosphodiester bridge in pseudotrisaccharide **370** [159]. The selectively protected glycoside **371** with a free hydroxyl group at C-4 (ManNAc) was transformed into phosphamidite **372** under the action of chlorophosphoramidite **24** (Scheme 30). The condensation of phosphamidite **372** with hemiacetal **373** resulted in the formation of a mixture of α- and β-glucopyranosides (α:β 55:45). The α-isomer was oxidized, decyanoethylated, and deprotected to obtain phosphodiester **370**.

Compounds **368**–**370** were printed on epoxysilane-coated slides together with bacterial *S. pneumoniae* 19A and *S. pneumoniae* 19F phosphoglycans as controls. A glycan microarray was arranged, and the glycan antigens interacted with the hyperimmune sera of rabbits immunized with whole bacteria. The group’s 19 sera were bound to *S. pneumoniae* 19A and *S. pneumoniae* 19F and phosphodisaccharide **368**, and the interaction with disaccharide **369** and the pseudotrisaccharide was weak. The interaction of synthetic and bacterial glycans was studied with factor reference antisera obtained by vaccination with whole cell bacteria *S. pneumoniae* 19A or *S. pneumoniae* 19F and cleared of the antibodies recognizing the common epitopes. Reference antisera to *S. pneumoniae* 19A readily bound to phosphate **368** and did not interact with compounds **369** and **370**. Reference antisera to S. pneumoniae 19F showed moderate binding to compounds **368**–**370**.

Two considered approaches to the search for a common epitope in *S. pneumoniae* 19A and *S. pneumoniae* 19F phosphoglycans open new horizons in the development of semisynthetic conjugated glycovaccines. The design of a universal glycotope for introduction in neoglycoconjugate vaccines is aimed at a decrease in protein loading of the vaccination dose and reduction of the production costs.

## 8. Synthesis of Pseudo-Oligosaccharides Structurally Related to Phosphoglycans of *C. Jejuni*

*C. jejuni* enteritis is one of the most common causes of intestinal infection and traveler’s diarrhea. It is associated with many severe sequelae, including Guillain–Barré syndrome. Penner serotyping in combination with PCR multiplex-based typing revealed 35 capsular types [160]. Until now, no commercial vaccines for the prevention of *C. jejuni* enteritis have been developed either for humans or for cattle and poultry, which are the frequent source of human campylobacteriosis. Whole-cell vaccines are not applicable, as a number of *C. jejuni* strains present sialylated LOS that induce antibodies to the gangliosides β-D-GalNAc-(1→4)-[α-Neu5Ac-(2→3)]-β-D-Gal-(1→4)-β-D-Glc-(1→1)-N-octadecanoyl sphingosine and α-Neu5Ac-(2→3)-β-D-Gal-(1→4)-β-D-Glc-(1→1)-N-octadecanoyl sphingosine, which are associated with the onset of Guillain–Barré syndrome [161]. Despite recent advances in the understanding of the pathogenesis of *C. jejuni*, the development of subunit or glycoconjugate preventive vaccines still remains a challenge [162]. Current knowledge on *C. jejuni* infection has revealed that the key structural feature is the O-methylphosphoramidate modification of capsular glycan, which is involved in both *C. jejuni* pathogenesis and immunogenicity. In the context of the development of conjugate vaccines on the basis of synthetic glycan antigens, O-methylphosphoramidate is regarded as the immunodominant epitope [163]. However, it should be noted that this moiety is easily hydrolyzed and can be lost during the conjugation process [160].

**Scheme 31:**
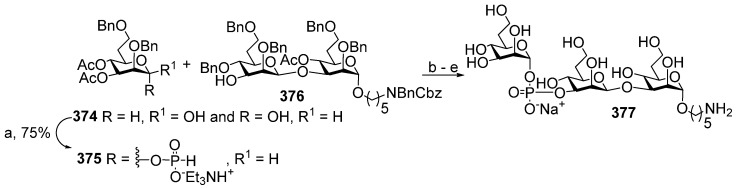
Key building blocks for the synthesis of capsular glucan of *C. jejuni* serotype HS:53, strain RM1221 [164]. Reagents and conditions: (**a**) chlorophosphite **129**, Py, MeCN, 0 °C → r.t.; (**b**) PivCl, Py, 1 h. 74%; (**c**) I_2_, Py/H_2_O, 0 °C. 0,5 h., 86%; (**d**) 1.2 M MeONa/MeOH; (**e**) H_2_/Pd-C, 48 h.

Up to now, only one synthesis of a P-linked pseudo-oligosaccharide related to *C. jejuni* phosphoglycans has been described. Therefore, the selectively protected hemiacetal **374** was converted into α-H-phosphonate **375** under the action of chlorophosphite **129**. The condensation of phosphonate **375** with the heptabioside block **376** in the presence of PivCl/Py, followed by oxidation, afforded a phosphodiester, which was then transformed into the target pseudo-trisaccharide **377** corresponding to a repeating unit of the capsular glycan of a clinically important strain RM1221 of *C. jejuni* serotype HS:53 (Scheme 31) [164]. The prospects of conjugated vaccines for the prevention of campilobacterioses are outlined in a recent review [165].

## 9. Conclusions

In this review, studies published over the last 20 years on the methods of construction of interglycosidic phosphodiester linkages used for the preparation of spacer-armed phosphooligosaccharides structurally related to the capsular phosphoglycans of the pathogenic bacteria *H. influenzae* serotypes a, b, c, and f, *N. meningitidis* serogroups a and x, *S. pneumoniae* serotypes 6a, 6b, 6c, 6f, 19a, and 19f, and the *C. jejuni* serotype HS:53, strain RM1221 are summarized.

As shown, in the syntheses of phosphoglycans, two types of intermediate compounds are widely employed: H-phosphonates and phosphoramidites. Both methods provide an effective tool for the establishment of phosphodiester bridges between different types of saccharide blocks. Due to high yields and simple procedures, these methods can be scaled up and form the basis for the manufacturing process. The H-phosphonate-based method is a one-pot polycondensation procedure used in the industrial-scale synthesis of Hib oligosaccharides for the manufacturing of the Quimi-Hib^®^ vaccine. However, the low stability of phosphodiesters in the conditions of the oxidation of interglycosidic phosphonates poses limitations on the preparation of oligomers with two or more phosphodiester bridges. The application of phosphoramidites is, in general, more efficient and allows the preparation of longer oligomers.

The majority of the spacer-armed synthetic fragments of phosphoglycans described in this paper were converted into neoglycoconjugates, which are shown to be safe and immunogenic in laboratory animals. For *Hib*, the combination of surface plasmon resonance, saturation transfer difference nanomagnetic resonance, and X-ray crystallography evidences that the minimal epitope consists of only two repeating units [29]. In the research conducted by Seeberger et al. [69], the conjugate with a tetramer *Hib* antigen (the shortest of 4, 6, 8, and 10-mers) was shown to be the most immunogenic. For Hia, the CRM197 conjugates of 1, 2, 3, and 4-mers were almost equally immunogenic. Immunogenicity with the conjugates of the phosphono-mimetics of *MenA* was independent of the length of the pseudo-oligosaccharide antigens, and non-acetylated carba-mimetics of *MenA* failed to evoke anti-*MenA* immunity. For *MenX*, immunization with conjugates of mono-, di-, and trimer with CRM197 was inefficient. For phosphooligosaccharides related to *S. pneumoniae* capsular phosphoglycans, the correlation between the length and immunity was not analyzed, as the majority of synthetic antigens comprised only one repeating unit. It can be concluded that longer oligomers are needed for a more profound study of immunogenicity, antigenicity, and protective immunity of neoglycoconjugates on the basis of synthetic phosphooligosaccharides.

Although the first examples of polysaccharide assembly via polycondensation (for example see [166]) were published almost 40 years ago, and in the last decade, synthesis of a high-molecular weight glycan containing 25-mer related to arabinogalactan [167] and a rhamnomannan consisting of 256 monosaccharide units [168], as well as an automated glycan assembly of a 151-mer polymannoside [169] and 15-mer phosphoglycan [50] related to the LPG of *Leishmania donovani* were reported, long oligosaccharides, which relate to bacterial phosphoglycans, are yet unattainable. However, the synthetic approaches described above permit the preparation of sufficiently long oligosaccharides of this type, including spacer-armed molecules, which are applicable as indispensable tools in any glycobiology studies and glycotechnology developments.

As described in this paper, a number of *Hib* and *MenA* oligomers were synthesized using a solid support method. This method has since evolved into a flexible and universal strategy based on the use of automated synthesis platforms. In glycochemistry, the Seeberger group [170] has been developing this approach for almost 30 years, and, finally, the automated glycan assembly technology has been created, which provides quick access to homogeneous oligomers and polymers starting from the properly protected monomer blocks [171]. Recent advances in automated glycan assembly [50,172,173] offer a powerful synthetic technique for the accomplishment of key steps in oligosaccharide synthesis. Protected monomer blocks required for automated glycan assembly can be attained through HPLC-based automated synthetic facilities [174,175,176].

As mentioned, *MenA* and *Hib* phosphoglycans are extremely susceptible to hydrolytic cleavage. For the preparation of stable *MenA* antigens, C-phosphono-mimetics were synthesized as non-acetylated compounds, and carba-mimetics were synthesized in both non-acetylated and acetylated variants. Immunization with corresponding protein conjugates was not efficient. It can be concluded that the problem of the preparation of a stable *MenA* antigen still waits to be resolved. On the contrary, Seeberger et. al. found that the introduction of a substituent, e.g., a methyl group, at O-2 provides a stable hydrolysis-resistant synthetic polyribosylribitolphosphate, which can be used in anti-Hib vaccines [77]. Also, the stability of an interglycosidic phosphodiester linkage can be achieved by the replacement of a non-bridging oxygen atom with a borano group [177].

To date, Quimi-Hib^®^ remains the only commercial vaccine with a fully synthetic phosphoglycan antigen. However, recent developments in glycobiology and glycochemistry provide new ways to the fundamental knowledge, which is pivotal for the design of new vaccine preparations on the basis of synthetic phosphooligosaccharides.

## Figures and Tables

**Table 1:** Synthesis of H-phosphonates and phosphoramidites from selectively protected 2-deoxy-2-azido-D-mannose and ManNAc hemiacetals.

Entry	Starting Hemiacetal (Even Numbers) and Target H-Phosphonate (Odd Numbers)	α-:β Ratio of Starting Hemiacetal	Reagents and Conditions	α-:β Ratio and Yield of the Target *H*-Phosphonate	References
1	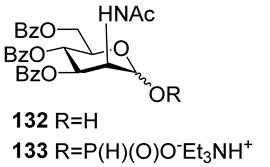	5:1	a, b *	5:1, 100%	[107]
2	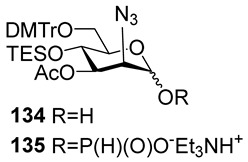	3.5:1	a, b *	3.5:1, 88%	[110]
3	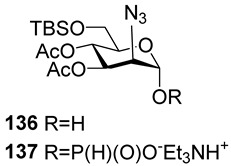	Only α	a, b *	Only α, 97%	[111]
4	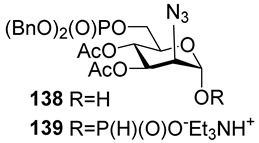	Only α	a, b *	Only α, the yield is not available	[111]
5	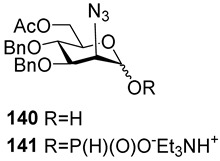	Only α	c, d *	93:3, 84%	[112]
6	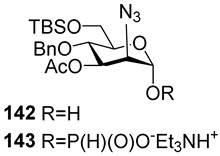	Only α	c, d *	Only α, 78%	[109]
7	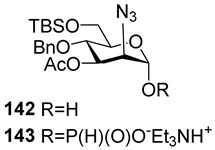	Only α	e, c *	Only α, 88–91%	[110,119]
8	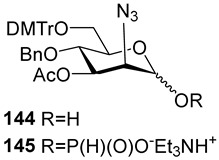	4.5:1	e, c *	Only α, 88%	[110]
9	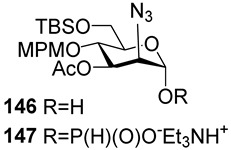	Only α	e, c *	Only α, 90%	[119]
10	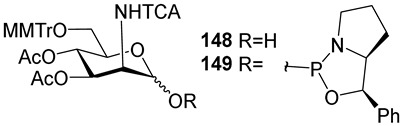	84:16	f *	Only α, 39%	[108]
11	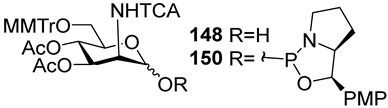	84:16	g *	Only α, 36%	[108]

* Reagents and conditions: (**a**) PCl_3_/imidazole, Et_3_N, MeCN, 0 °C; (**b**) 0.5 M aq. Et_3_NH∙HCO_3_, pH 7–8; (**c**) 7 eq. (PhO)_2_P(O)H, Py, 84%; (**d**) 50% (vol.) Et_3_N aq. solution; (**e**) **129** (Figure 8), Et_3_N, dioxane, 0 °C; (**f**) **130** (Figure 8), 3 eq, Et_3_N, 7 eq, THF, −78 °C→r.t., 3 h; (**g**) **131** (Figure 8), 3 eq, Et_3_N, 7 eq, THF, −78 °C→r.t., 3 h.

## Abbreviations

The following abbreviations are used in this manuscript:

avDP	average degree of polymerization
Ac_2_O	acetic anhydride
Boc	tert-butyloxycarbonyl
BOM	benzyloxymethyl
CA	chloroacetyl
Cbz	benzyloxycarbonyl
CPS	capsular polysaccharide
CRM197	cross-reacting material 197, a non-toxic mutant of the diphtheria toxin
DBU	1,8-diazabicyclo(5.4.0)undec-7-ene
DCI	4,5-dicyanoimidazole
DCSO	(+)-(10-camphorsulfonyl)oxaziridine
DIAD	diisopropyl azodicarboxylate
DIC	1,3-diisopropylcarbodiimide
DIPEA	N,N-diisopropylethylamine
DMTr	bis(4-methoxyphenyl)-phenylmethyl
DMAP	4-dimethylaminopyridine
DMSO	dimetylsulfoxide
DMTr	4,4-dimethoxytrityl
EC50	half maximal effective concentration
ELISA	enzyme-linked immunosorbent assay
EtOAc	ethyl acetate
EtOH	ethanol
HSA	human serum albumin
IC	inhibitory concentration
IgA	immunoglobulin A
IgG	immunoglobulin G
IgM	immunoglobulin M
KLH	keyhole limpet haemocyanin
mCPBA	3-chloroperbenzoic acid
*MenA*	*Neisseria meningitidis* serogroup a
*MenX*	*Neisseria meningitidis* serogroup x
MMTr	monomethoxytrityl
MPM	4-methoxybenzyl
Nap	2-naphthylmethyl
MeOPN	O-methylphosphoramidate
OS	oligosaccharide
PBS	phosphate buffer saline
PCV	pneumococcal conjugated vaccines
PEG	polyethylene glycol
PivCl	pivaloyl chloride
Ph_3_P	triphenylphosphine
PhSH	thiophenol
PMP	4-methoxyphenyl
pNP	4-nitrophenyl
PRP	poly-3-β-D-ribosyl-(1→1)-D-ribitol-5-phosphate
Py	pyridine
rSBA	bactericidal assay (SBA) titers using rabbit serum
SMP	succinimidyl-3-maleimidopropionate
TBDPS	tert-butyldiphenylsilyl
TDS	thexyldimethylsilyl
TCA	trichloroacetic acid
TES	triethylsilane
TFA	trifluoroacetic acid
THF	tetrahydrofuran
TT	tetanus toxoid
